# Generation and characterization of early stage oral cancer cell line of buccal mucosa of Indian origin

**DOI:** 10.1007/s13577-025-01332-6

**Published:** 2025-12-18

**Authors:** Akhila George, Sudhir Nair, Kumar Prabhash, Sayujata Thakur, Poonam Gera, Arjun Singh, Pankaj Chaturvedi, Swapnil Rane, Trupti Pradhan, Subrata Sen, Madan Barkume, Dhanlaxmi Shetty, Kruti Chaubal, Arpita Ghosh, Sanjeev Kamte, Jyoti Anand Kode

**Affiliations:** 1 Kode Lab, Tumor Immunology and Immunotherapy (TII) Group, Advanced Centre for Treatment, Research and Education in Cancer (ACTREC), Tata Memorial Centre, Kharghar, Navi Mumbai, 410210 India; 2 Homi Bhabha National Institute (HBNI), Training School Complex, Anushakti Nagar, Mumbai, 400094 India; 3 Department of Surgical Oncology, Tata Memorial Hospital, Tata Memorial Centre, Mumbai, 400012, India; 4 Head and Neck Disease Management Group, Tata Memorial Hospital, Tata Memorial Centre, Mumbai, 400012, India; 5 Department of Medical Oncology, Tata Memorial Hospital, Tata Memorial Centre, Parel, Mumbai, 400012 India; 6 Homi Bhabha Cancer Hospital & Research Centre (HBCH&RC), Tata Memorial Centre, Muzaffarpur, Bihar 842004, India; 7 Biorepository Facility, ACTREC, Tata Memorial Centre, Kharghar, Navi Mumbai, 410210, India; 8 Department of Pathology and Digital and Computational Oncology, ACTREC, Tata Memorial Centre, Kharghar, Navi Mumbai, 410210, India; 9 Anti-Cancer Drug Screening Facility (ACDSF), ACTREC, Tata Memorial Centre, Kharghar, Navi Mumbai, 410210, India; 10 Department of Cancer Cytogenetics, ACTREC, Tata Memorial Centre, Kharghar, Navi Mumbai, 410210 India; 11 Eurofins Genomics India Pvt. Ltd, Bengaluru, Karnataka India

**Keywords:** Cell line, Early-stage buccal mucosa cancer, Pre-clinical screening tool, NLRP3 therapeutic target, Chemosensitivity, Cell xenograft, Generation and establishment of cell line

## Abstract

**Abstract:**

Being topmost cancer in India, oral cancer management warrants discovery of novel biomarkers, treatment strategies, and targets to help with early diagnosis, treatment, and recovery. To have a continuous supply of cells, the study was aimed at generation and characterization of established cell line from buccal mucosa (BM) tumors from patients of Indian origin which can be developed as a pre-clinical tool for biomedical application. Surgically resected tumor tissue from histo-pathologically confirmed oral cancer were processed for explant culture. TBM-02 cell line was passaged and characterized for morphology and function. Further, the cell line was silenced for inflammasome pathway gene NLRP3 to evaluate its linkage with oral cancer tumorigenesis. TBM-02, successfully established from BM, was maintained up to 100 passages, exhibited epithelioid morphology, high EpCam expression and triploid ploidy with chromosomal aberrations. Novelty and human origin of TBM-02 was authenticated by Short Tandem Repeats profiling and comparison with DSMZ database. TBM-02 revealed tumorigenic potential in vitro and in vivo which was abrogated on silencing NLRP3. Increased expression of NLRP3, hallmark of chronic inflammation in TBM-02, was validated at protein and gene level and in xenograft. TBM-02 demonstrated migratory potential and was found to be a sensitive tool to study drug response. RNA sequencing demonstrated upregulation of oral cancer-associated genes and pathways. Thus, in current study, we have reported development of novel cell line from early-stage buccal mucosa cancer patient which has a strong potential to be developed and to be used as pre-clinical model for improving oral cancer management and therapeutics.

**Graphical Abstract:**

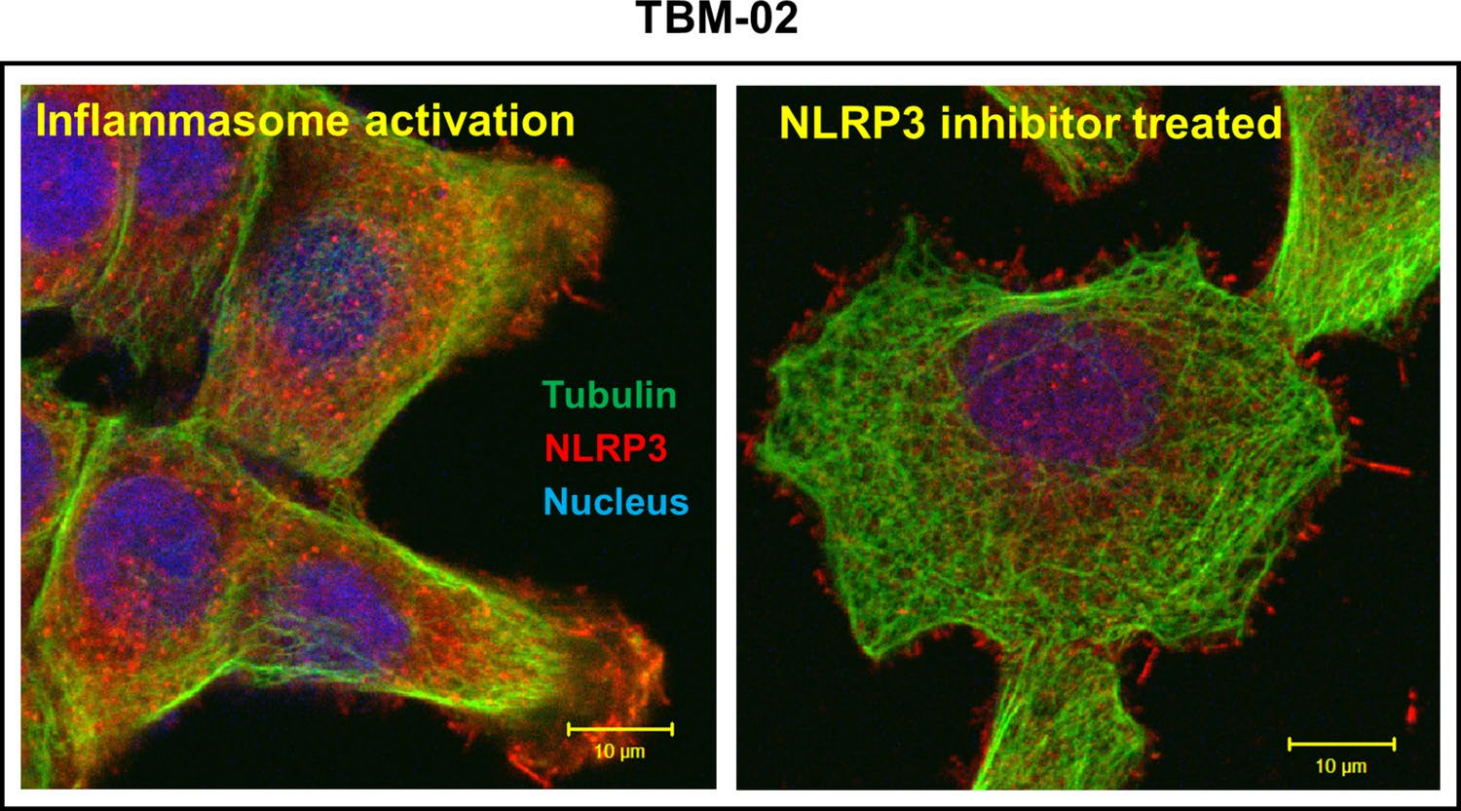

**Supplementary Information:**

The online version contains supplementary material available at 10.1007/s13577-025-01332-6.

## Introduction

Oral squamous cell carcinoma (OSCC) is the second most prevalent cancer in India, predominantly affecting men. Global mortality rate has shown 1.4-fold increase since the past 3 decades [1], a 5-year overall survival of 65% [2]. Gingivobuccal cancers are the Indian type of cancer attributed to smokeless tobacco chewing habits in India. It involves the cancer of buccal mucosa, gingivobuccal sulcus, lower gingiva, and retromolar trigone [3]. The risk factors include tobacco consumption in the form of bidi smoking, smokeless tobacco habits like paan, betel quid chewing with areca nut, etc. Alcohol and HPV are the other two independent factors known to cause oral cancer [4].

Despite being at an easily accessible site, oral cancer is often presented at late stages of disease progression [5]. There is a need to identify molecular drivers, molecular mechanism of tumor heterogeneity, de novo or acquired resistance and so on. Appropriate models are also required for screening various potential drug candidates.

Models are a necessary requirement to study any disease pathology. Appropriate models would help to understand the disease better and helps to shape a therapeutic strategy in a very streamlined manner early on [6]. Cell lines offer numerous advantages in the field of cancer research, particularly in understanding the intricate pathways within cells. Moreover, utilizing cell lines reduces time constraints and facilitates easier maintenance. This not only streamlines research processes but also aids in effective resource management. It would be most appropriate to develop efficacious drugs against oral cancer within India using pre-clinical models developed from oral tumors of Indian origin. Few groups from India have successfully reported development of pre-clinical cell lines from buccal mucosa at advanced stages [7, 8].

We hypothesize that if we are able to effectively treat oral tumors at the early stages, we will be able to control the disease without compromising the quality of life of patients. Hence, to screen and develop drugs which act on the early stage oral cancer, our current study was aimed at establishing pre-clinical model using early stage buccal mucosa oral tumor.

Chronic inflammation is one of the hallmarks of cancer. Central to this is cytoplasmic multiprotein complex called “NLRP3 inflammasome” which orchestrates innate immune mechanisms to regulate homeostasis or progression to cancer [9]. There have been reports of activation of inflammasome assembly using activators and stimulators like lipopolysaccharide, nigericin, gramicidin, and ATP [10]. Therefore, our consequent aim was to establish inflammasome activation model of early stage buccal mucosa cancer. There are no earlier reports of such inflammasome activation model in oral cancer cell lines of Indian origin.

NLRP3 inflammasome has been shown to be a therapeutic target and directly associated with tumor initiation, promotion, and progression due to high expression of NLRP3 and IL-1β in OSCC tissue [11]. Another study demonstrated that knockdown of NLRP3 led to reduced xenograft tumor growth in prostate cancer in in vivo model [12]. Hence, our next aim was to create a NLRP3 knockdown model of early stage oral cancer cell line.

Thus, our current study involves generation and characterization of early stage buccal mucosa oral cancer cell line, inflammasome activation model, and NLRP3 knockdown model of this cell line which can have immense applications for screening and developing therapeutic drugs for improved oral cancer management.

## Materials and methods

### Study subjects

Surgically resected fresh tumors of OSCC of buccal mucosa (*n* = 10) were collected from treatment naïve patients who were clinically and histo-pathologically confirmed with diagnosis of OSCC. The tumor size ranged from 50 to 100 mg. All the experimental procedures and tumor sample collection were conducted as per guidelines issued by Institutional Human Ethics Committee (IEC-III, approval #900838). The patient OC-P2 from whom TBM-02 was developed was a 34-year male with a history of chewing tobacco and confirmed pT2N0 according to AJCC TNM staging (8th Edition).

### Cell lines and reagents

AW13516 tongue cancer cell line indigenously developed from Indian patient in our department was used for comparison control [13]. HEK293FT was provided by Dr. Didier Trono, École Polytechnique Fédérale de Lausanne, Switzerland. The media used for tumor tissue collection and transport consisted of plain Iscove’s modified Dulbecco’s media (IMDM), supplemented with 8% antibiotic cocktail. Enzyme cocktail for tissue disruption consisted of collagenase, hyaluronidase, and DNAse. For culturing cells, plain IMDM was supplemented with 10% fetal bovine serum (FBS), L-glutamine (2 mM), and 4% antibiotic cocktail. For characterization, either purified or fluorescent-conjugated antibodies against human markers viz. Epithelial Cell Adhesion molecule (EpCAM), α-SMA (α-smooth muscle actin), Nucleotide-binding domain (NOD)-, leucine-rich repeat (LRR)-, and pyrin domain (PYD)-containing protein 3 (NLRP3), IL-18, Gasdermin-D (GSDMD), Caspase-1 and α-Tubulin were used. For chemosensitivity assay, one phytochemical 6-shogaol and standard chemotherapeutic drugs viz. gemcitabine, cisplatin, carboplatin, mitoxantrone, etoposide, oxaliplatin, vinblastine, paclitaxel, and doxorubicin were used. Details of reagent and buffers are provided in supplementary methods and Supplementary Table S1.

### Tumor processing and explant culture

The buccal mucosa tumor tissue in transport media was transferred to tissue culture laboratory on ice as early to avoid loss due to degradation. The resected tumor tissue was first rinsed with 2% betadine, necrotic tissue and blood clots were also removed. In brief, the tumor tissue was finely cut into small pieces and enzymatically digested followed by crushing with a sterile piston and passing through 40-μM nylon cell strainer. The viability of the cells was obtained by trypan blue dye exclusion staining. The cells were plated to 60-mm cell culture Petri plates and incubated at 37 °C in CO_2_ incubator. The cells were observed regularly under bright field microscope for contamination and growth. After 2 days, the floating dead cells were removed by a phosphate buffered saline (PBS) wash. The cells began to appear 2–3 days after plating. The cultures were maintained for 2–3 weeks by replenishing with fresh complete IMDM twice a week.

### Subculture and cryopreservation of cell line

After 2–3 weeks, the TBM-02 cells grew to a sufficient confluency and were sub-cultured in 25 cm^2^ flask using 0.3% trypsin–EDTA prepared in PBS. Confluent cells were obtained after 1–2 weeks of culture. The cell line has been sub-cultured up to 100 passages. At every passage, the cells have been cryopreserved in liquid nitrogen for future use. Cultured cells in log-phase were pelleted by centrifugation at 1000 rpm. The pellet was resuspended in cold freezing mixture with 10% dimethyl sulfoxide (DMSO) and 90% FBS. The freezing mixture was added slowly with constant mixing, and the suspension was transferred to sterile freezing vials and stored in liquid nitrogen for long-term storage.

### Mycoplasma detection

To ascertain if cells TBM-02 (passage 92) were free of mycoplasma contamination, cell supernatant was tested using Mycoplasma Detection Kit-Quick test (Biotool.com; Cat. No. B39038). The supernatants of cultures of older than 48 h were used to check the presence of mycoplasma contamination according to manufacturer’s protocol. Detailed protocol is provided in supplementary file.

### Molecular and morphology characterization

#### Genomic fingerprinting by short tandem repeat (STR) analysis

STR profiling of TBM-02 (Passage 50) was conducted as described previously [14]. Eighteen STR loci were amplified using 18D PowerPlex kit (Promega, USA), and the products were subjected to capillary electrophoresis 3500 Genetic Analyzer (Applied Biosystems, USA). Data were analyzed using SoftGenetics (Gene Marker, USA). Appropriate controls were included for each sample run. STR profile/genotyping data of the tested sample are queried for a profile match with the DSMZ database that catalogs cell line STR profiles.

#### Karyotyping and ploidy analysis

The chromosome profiling of TBM-02 was done by karyotyping at passage 5 and 67 to assess tumor cell heterogeneity and chromatin stability upon passaging. The cells were cultured, grown to appropriate confluency, and treated with colchicine for 17 h to arrest cell cycle at metaphase. Detailed protocol is provided in the supplementary file.

To determine DNA ploidy, TBM-02 cells at passages 14 and 52 were seeded at a density of 0.1 × 10^6^ in 6 well plates for 48 h in CO_2_ incubator. After 48 h, the cells were trypsinized (0.3% Trypsin–EDTA), washed and fixed with 70% chilled ethanol. The cells were treated with 100 μg/mL RNase A and stained with 40 μg/mL propidium iodide. The cells were incubated at 37 °C for 30 min. The DNA content was analyzed by acquiring cells on Attune NxT flow cytometer (ThermoFischer, USA) and analyzed using ModFit software (Version 5.0, Verity, USA).

#### Ultrastructural morphology by transmission electron microscopy

To understand ultrastructural morphology, cells were observed under electron microscopy. Cells were seeded at a density of 0.1 million in a 35-mm Petri plate and grown to a confluency of 80%. The cells were fixed with glutaraldehyde and post-fixed with osmium tetroxide Electron micrographs were captured by JEOL JEM 1400 Plus Transmission Electron Microscope (JEOL, Japan) at 120 kV. Detailed protocol is provided in supplementary methods.

#### Epithelial phenotype assessment of TBM-02 by flow cytometry

To determine the squamous epithelial origin of the established cell line, the cells were stained with EpCAM which is a marker of rapidly proliferating epithelial tumors, and to rule out fibroblast contamination, the cells were further stained using antibodies against fibroblast marker α-SMA and epithelial cell marker EpCAM, and incubated for 45 min at 4 °C. The cells were washed with FACS buffer and acquired on flow cytometer (FACS ARIA III, BD Biosciences, USA).

### Functional characterization

#### Growth kinetics

To assess growth kinetics, cells were seeded in 60-mm Petri plate at a density of 0.1 × 10^6^ and incubated at 37 °C in CO2 incubator. The cultures were harvested at 12, 24, 48, 72 and 96 h. The cell count was determined by staining them with 0.4% trypan blue dye by dye exclusion method. The doubling time was calculated using the formula *T*. In2/In (*Xe*/*Xb*) where T = time of incubation, Xe = no. of cells at the end of incubation, and Xb is the number of cells the beginning of incubation.

#### Colony formation assay

To check the clonogenicity of TBM-02 cells, we performed a colony formation assay. Cells were seeded at a density of 1500 cells per well to allow for colony formation for 7 days. Cells were fixed by adding 2 mL of 4% PFA (Paraformaldehyde) solution to each well and incubated for 15 min at room temperature. PFA was removed and wells were washed with 1 × PBS. Colonies were stained using crystal violet, counted and recorded the number of colonies by ImageJ software.

#### Trans-well migration assay

Cellular migration was assessed using migration assay. 8-μM pore size polycarbonate fiber inserts with 24-well companion chamber (Boyden chamber) were used for migration assay. 10,000 cells in 100 μL plain IMDM were seeded onto the membrane of trans-well insert. Medium containing 10% FBS was used as chemoattractant and was carefully added below the trans-well insert and incubated for 24 h. After incubation, the trans-well insert was removed carefully with forceps. The non-migrated cells were removed from the apical side of the insert using a cotton-tipped applicator. The migrated cells on the basal side of the membrane were fixed with 100% methanol and stained with 0.2% crystal violet. The migrated cells were observed as images with microscope.

#### Transcriptome analysis

Total RNA was isolated from the TBM-02 (p11 and p62) cell line using Quick RNA MiniPrep Plus kit (Zymo Research) as per manufacturer’s protocol. The quality and quantity of the RNA were determined by Nanodrop followed by agilent tape station using high-sensitivity RNA screen tape. The RNA-Seq paired end sequencing libraries were prepared using Illumina TruSeq mRNA sample prep kit. The libraries were enriched by limited number of PCR cycles, purified and were checked for quantity and quality. Cluster generation and sequencing was done by loading the PE Illumina libraries onto Novaseq6000 platform. For normal buccal mucosa, the SRA files were downloaded from the SRA database (SRX19125628 and SRX19125629) and were subsequently converted into raw fastq files using fastq-dump. The detailed protocol is provided in the supplementary file.

#### Evaluation of tumorigenicity potential

##### Xenograft induction and characterization

NOD-SCID mice were maintained in laboratory animal facility (ACTREC) which was used for in vivo tumorigenesis studies. All procedures involving mice were performed according to the protocols approved by the Institutional Animal Ethics Committee, Tata Memorial Centre, Navi Mumbai (Proposal # 16/2021 and 23/2024) and were adhered to the CCSEA guidelines (Registration Number: 65/GO/ReBiBt/S/99/CCSEA). 6–8-week-old male mice weighing 18–22 g were used for all the experiments. TBM-02 cell suspension was injected subcutaneously into the flank of the mice (*n* = 2) using 1 mL syringes with 25G needles. Mice were monitored every 3 days for body weight, tumor volume, and mortality. Mice were observed for tumor for 40 days. Tumor volume was calculated using the formula for the volume. The tumor volume was calculated using the formula *w*1 × *w*1 × *w*2 × *π*/6 (*w*1—smallest width, *w*2—largest width). Details are provided in supplementary file.

##### Immunohistochemistry of patient-derived xenograft

IHC was performed as described previously by Shi et al. [15]. Briefly, the tissues were deparaffinized, rehydrated, and antigen retrieval was done by placing the slides in antigen retrieval buffer at 98 °C in a water bath. The slides were blocked and then incubated with primary antibody at 4 °C overnight. Next day, the slides were washed and incubated with horseradish-peroxidase conjugated respective secondary antibodies for 20 min. Then the slides were stained with DAB followed by counterstain with hematoxylin and scored by pathologist.

### NLRP3 inflammasome activation

To study the NLRP3 inflammasome pathway, TBM-02 cells were seeded at appropriate seeding density and allowed to adhere overnight. The cells were then treated with NLRP3 inhibitor 5 µM MCC 950 or 5 µM and 10 µM 6-Shogaol for 1 h before priming with 500 ng/mL LPS for 12 h. After priming, the cells were then treated with 5 µM Nigericin for 1 h which is a known activator of NLRP3 pathway. After activation, the cells were harvested or fixed for respective assays.

#### Validation using RT-PCR

The treated cells were harvested, fixed, and stored in TRIzol at − 80 °C. RNA isolation was carried out using manufacturer’s protocol. cDNA was synthesized from the RNA using RevertAid H minus First strand cDNA synthesis kit following the manufacturer’s protocol. Further, real-time PCR was carried out using KAPA SYBR FAST Universal. The reaction system and cycling conditions were set according to the manufacturer’s instructions. The primers’ details are provided in Supplementary Table S2.

#### Immunofluorescence and confocal microscopy

5000 TBM-02 cells were seeded in glass bottom Petri plate overnight. For inflammasome activation, the cells were treated with the inhibitor for 1 h, then they were primed with 500 ng/mL of LPS overnight. Next day, the cells were treated with 5 μM Nigericin for 1 h. After incubation, the cells were washed and fixed with 4% PFA for 15 min at room temperature. The cells were permeabilized and blocked with 0.1% NP-40 and 3% BSA, respectively. The cells were then labeled with respective primary antibody overnight and then with the corresponding secondary antibody for 45 min at room temperature. The cells were stained with DAPI for nuclear staining and images were captured using LSM 980 confocal microscope**.** Expression of cytokeratin 8 and cytokeratin 14 in TBM-02 was also checked by immunofluorescence for confirming epithelial origin.

### Development of in vitro gene silencing model

In vitro knockdown of NLRP3 gene in TBM-02 using shRNA-mediated gene silencing was carried out using pLKO_005 carrying NLRP3 shRNA (short hairpin RNA) which was procured from Merck (Sigma Aldrich cat # TRCN0000419896). Next, the selected shRNA constructs were transduced using lentivirus-mediated transduction method for stable integration into the genome of TBM-02 oral cancer cell line. The detailed protocol is provided in supplementary methods.

### Scope of application

#### Pre-clinical model to test drug efficacy

Sensitivity of the cell line to various commercially available chemotherapeutic drugs was also evaluated by Sulphorhodamine-B assay. The cells were seeded at a density of 2000 cells per well in 96 well plates. After 24 h, the cells were treated with appropriate concentration of known drugs and incubated for 48 h. After 48 h, the cells were fixed with 10% trichloroacetic acid at 4 °C for 1 h. The plates were washed with slow running tap water four times and air dried. After drying, the plates were stained with 0.057% SRB for 30 min at room temperature and rinsed with 1% acetic acid to wash off excess dye. The plates were then air dried and the dye was solubilized using 10 mM Tris-base. The optical density was measured at 510 nm. The IC_50_ was determined using curve-fitting method.

## Results

### Cell line generation and maintenance

**Fig. 1:**
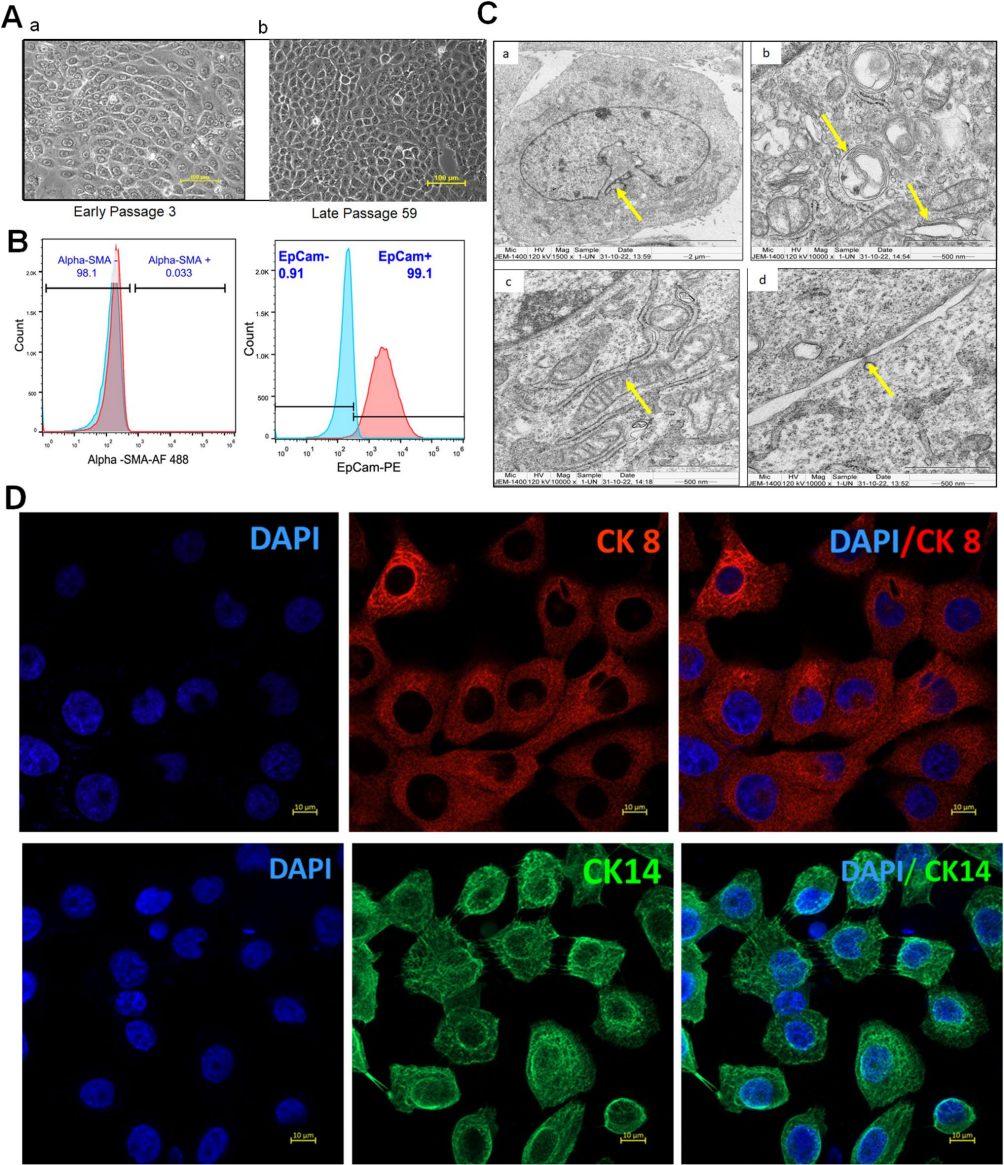
Morphologic and phenotypic characterization of TBM-02. Panel A gives bright field microscopy view of TBM-02 at early passage 3(a) and late passage 59(b). Panel B demonstrates phenotypic profile of TBM-02 by flow cytometry for surface expression of α-SMA (**a**) and EpCam (**b**). Blue histogram denotes control and red histogram denotes marker expression. Ultrastructural morphology of TBM-02 is depicted in panel C. The arrows indicated invaginating nuclear membrane (**a**), autophagy vesicles and elongated ER (**b**), mitochondria with distinct cristae (**c**) and desmosomes (**d**). Panel D exhibits Cytokertain 8 (CK8, Red) and Cytokeratin 14 (CK 14, Green) expression by immunofluorescence and laser confocal microscopy. Nucleus is stained blue by DAPI. Scale bar: 10 μm

Of the samples studied, the cell line could be generated from subject # OC-P2. The clinicopathological details of the patients are given in Supplementary Table S3. The cell line was established from 34-year-old, male patient with a history of chewing tobacco. The tissue was histopathologically confirmed to be pT2 pTN0 with moderately differentiated squamous cell carcinoma. Our study is the first to establish and characterize a cell line of early-stage buccal mucosa cancer of Indian patient. We have compiled data of all the cell lines of buccal mucosa of Indian and International origin comparing the clinicopathological features in Table 1. The explant cultures demonstrated cell growth as early as 2nd day. The cells had typical epithelioid morphology (Fig. 1A) with high nuclear to cytoplasmic ratio and consisted of giant multinucleated cells and successfully cultured upto100 passages. It exhibited epithelial cancer-associated marker EpCAM (99.1%) and was devoid of fibroblast specific marker α-SMA (0.033%) (Fig. 1B). The cell line also stained positive for epithelial markers cytokeratin 8 and cytokeratin 14 (Fig. 1D).

**Table 1:** Description of buccal mucosa cell lines

	Cell line name		Age, gender	Ethnicity	Primary site	Staging	Histology (grade)	Habits	References
1*	ITOC 1		28, male	India	Buccal mucosa	pT4 N2c M0	SCC (poorly differentiated)	Chronic tobacco chewer	[8]
2	ITOC 3		37, male	India	Buccal mucosa	pT3 N2b M0	SCC (poorly differentiated)	Chronic tobacco chewer	[8]
3	ITOC 4		61, male	India	Buccal mucosa	pT4a N2b M0	SCC (poorly differentiated)	Chronic tobacco chewer	[8]
4	ACOSC 3		40, male	India	Buccal mucosa	pT2 N2 M0	SCC (moderately differentiated)	Chronic tobacco chewer	[7]
5	ACOSC 4		70, female	India	Buccal mucosa	pT4 N2b M0	SCC (moderately differentiated)	Chronic tobacco chewer	[7]
6	ACOSC 16		34, male	India	Buccal mucosa	pT4a N2b M0	SCC (moderately differentiated)	Chronic tobacco chewer	[7]
7	TBM-02		34, male	India	Buccal mucosa	pT2 N0	SCC (moderately differentiated)	Chronic tobacco chewer	
8**	OC-2		51, male	China, Taiwan	Buccal mucosa	Not available	SCC	Smoking and Chronic tobacco chewing	[16]
9	OC-3		57, male	China, Taiwan	Buccal mucosa	Not available	Not available	Areca-nut chewing and drinking	[17]
10	OCC-05		63, female	Iran	Buccal mucosa	T1N0	SCC (well differentiated)	No smoking	[18]
11	OCC-27		55, female	Iran	Buccal mucosa	T2N0	SCC (moderately differentiated)	Non-smoker	[18]
12	OCC-28		59, male	Iran	Buccal mucosa	T1N0	SCC (well differentiated)	Smoker	[18]
13	OCC-32		55, female	Iran	Buccal mucosa	Not available	SCC (moderately differentiated)	Not available	[19]
14	OCC-34		60, male	Iran	Buccal mucosa	Not available	SCC (well differentiated)	Not available	[19]
15	ORL-195		61, female	Indian	Buccal mucosa	T2N0	Not available	Betel quid chewer	[20]
16	ORL-196		59, female	Indian	Buccal mucosa	T2N2	Not available	Betel quid chewing and alcohol	[20]
17	ORL-204		76, male	Indian	Buccal mucosa	T2N1	Not available	Tobacco, betel quid, and alcohol	[20]
18	ORL-214		49, female	Indian	Buccal mucosa	T4N0	Not available	Betel quid chewer	[20]
19	UPCI-SCC-029A		84, male	Caucasian	Buccal mucosa	T4N2	Not available	Not available	[21]
20	USC-HN2		81, female	Caucasian	Buccal mucosa	T4N0M0	SCC (moderately to poorly differentiated)	Tobacco smoking and alcohol consumption	[22]
21	UT-SCC-122		36, male	Caucasian	Buccal mucosa	T3N0M0	Grade 2	Not available	[23]
22	UT-SCC-54A		58, female	Caucasian	Buccal mucosa	T2N0M0	Grade 1	Not available	[23]
23	VU-SCC-1365		22, male patient diagnosed with Fanconi’s anemia	Paris	Buccal mucosa	unknown	Not available	Not available	[24]
24	YD-9	56, male		China	Buccal mucosa	unknown	Moderately differentiated squamous cell carcinoma	Not available	[25]

*S. No. 1–7 are the cell lines established in India

**S. No. 8–24 are the cell lines established outside India

At ultrastructural level, the cells contain large nucleus with deep indentation. They have long mitochondria with distinct cristae, desmosomes, and microfilaments also (Fig. 1C). Dilated endoplasmic reticulum was visible. Doubling time of TBM-02 was found to be 26.39 h (Supplementary Fig. S1).

### Karyotype and DNA ploidy analysis

**Fig. 2:**
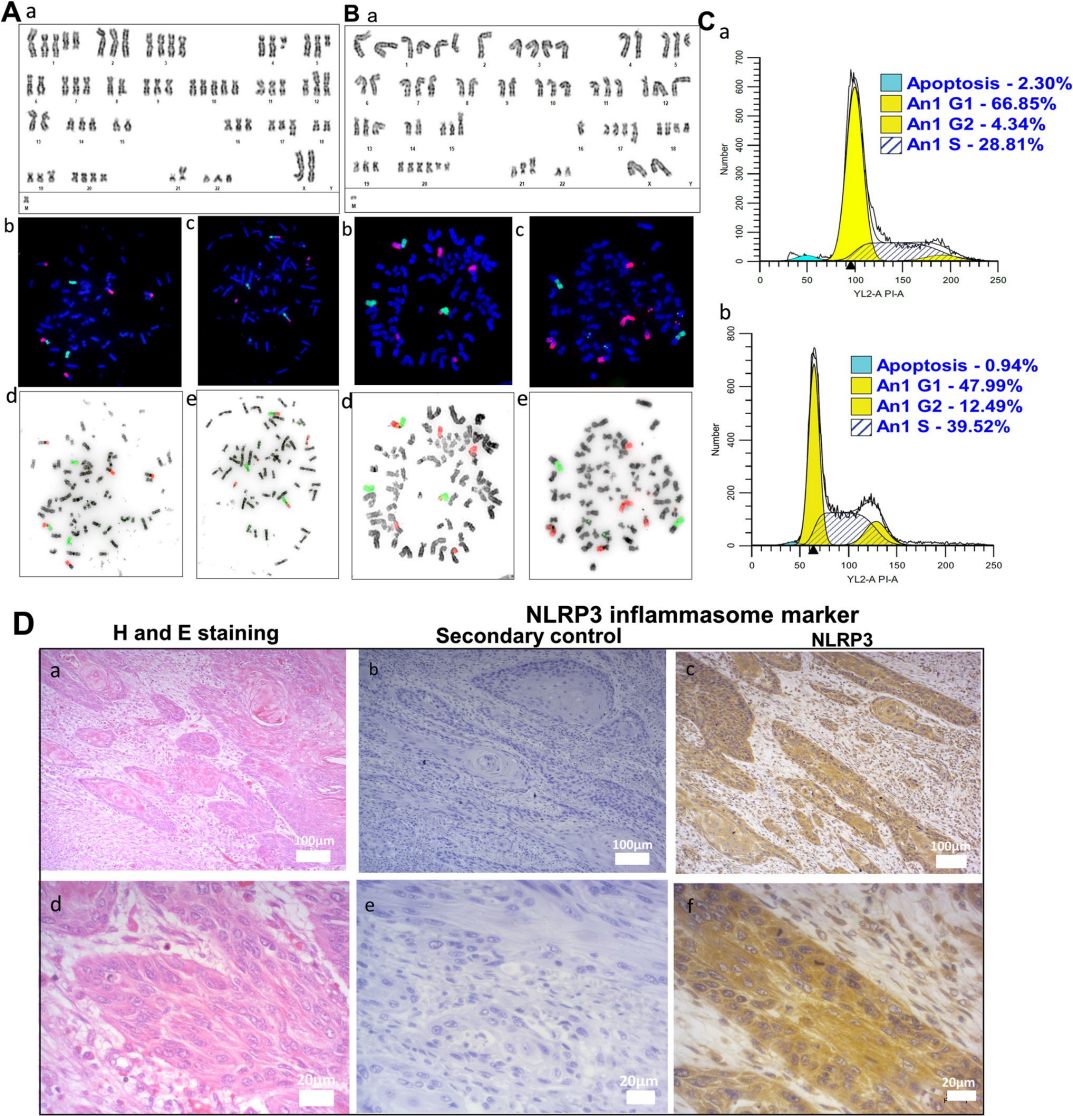
Karyotyping and STR profiling of TBM-02. Details of karyotyping of TBM-02 cells at passage 5 (A) and passage 67 (B) are given as conventional composite karyotype (subpanels Aa and Ba), Metaphase FISH using WCP 10 (green) and WCP 13 (red) (subpanel Ab), WCP 12 (green) and WCP 15 (red) (subpanel Ac), Metaphase FISH using whole chromosome paint probes WCP 10 (green) and WCP 13 (red) (subpanel Bb), Metaphase FISH using whole chromosome paint probes WCP 1 (red) and WCP X (green) (subpanel Bc), subpanels Ad, Ae, Bd, and Be are inverse DAPI of Ab, Ac, Bb, and Bc, respectively. Panel C depicts cell ploidy analysis of TBM-02 at passages 14 (**a**) and passage 52 (**b**). The legend in the top right represents percent population in the respective cell cycle phases. Panel D demonstrates the histology of the tumor tissue of oral cancer patient from which TBM-02 was derived and the expression of NLRP3 protein in the tumor tissue. Sections represent H and E-stained tumor tissue (Da and Dd), secondary control-stained sections (Db and De), and immunohistochemistry stained for NLRP3 inflammasome marker expression (Dc and Df). Magnifications are 10 × (Da, Db, and Dc) and 40 × (Dd, De, and Df)

The karyotyping of TBM-02 at early passage P5 (Fig. 2A) and late passage P67 (Fig. 2B) revealed triploidy with 69 chromosomes. Various numerical and structural aberrations with additional abnormalities observed in P67 as compared to P5 are: addition of der(1)ins(1;13)(p11;q14q34) and isochromosome 1, deletion of chromosome 2, deletion of der(4)del(4)(q11), additional chromosome 8, deletion of extra chromosome 9, additional chromosome 13, deletion of extra chromosome 14, deletion of chromosome 16, additional chromosome 17, addition of two derivative chromosome 18, addition of two derivative chromosome 20, addition of derivative chromosome 21, and deletion of extra chromosome 22. Loss of Y chromosome was observed in both the passages (Fig. 2A, B). Cell cycle analysis revealed that TBM-02 cells possess a complex and consistent aneuploid nature at various passages with DNA index of 2.242 (passage 14), 1.44 (passage 52), and 1.77 (passage 62, 64, and 65) (Fig. 2C-a, C-b). H and E staining of the original tumor tissue resected from patient shows moderately differentiated squamous cells carcinoma (Fig. 2D-a, Dd). These sections also had distinct expression of NLRP3 protein in cytoplasm of tumor cells (Fig. 2D-c, D-f).

Authenticity of the cell line was validated by STR analysis, and TBM-02 does not show a match with any cell line cataloged in DSMZ database. Y allele was not detected in the amelogenin marker in STR analysis (Table 2). Cell line culture supernatant at passage p92 was found to be negative for mycoplasma. (Supplementary Fig. S2).

**Table 2:** STR analysis of TBM-02 at passage 50

STR locus	Alleles
D3S1358	16, 18
THO1	9
D21S11	28.2, 29.2
D18S51	16
Penta E	7, 11
D5S818	10, 12
D13S317	13
D7S820	10, 13
D16S539	12
CSF1PO	11, 12
Penta D	11, 14
Amelogenin	X
vWA	15, 18
D8S1179	10, 16
TPOX	11
FGA	24.2
D19S433	13.2
D2S1338	16, 19

### Functional characterization

**Fig. 3:**
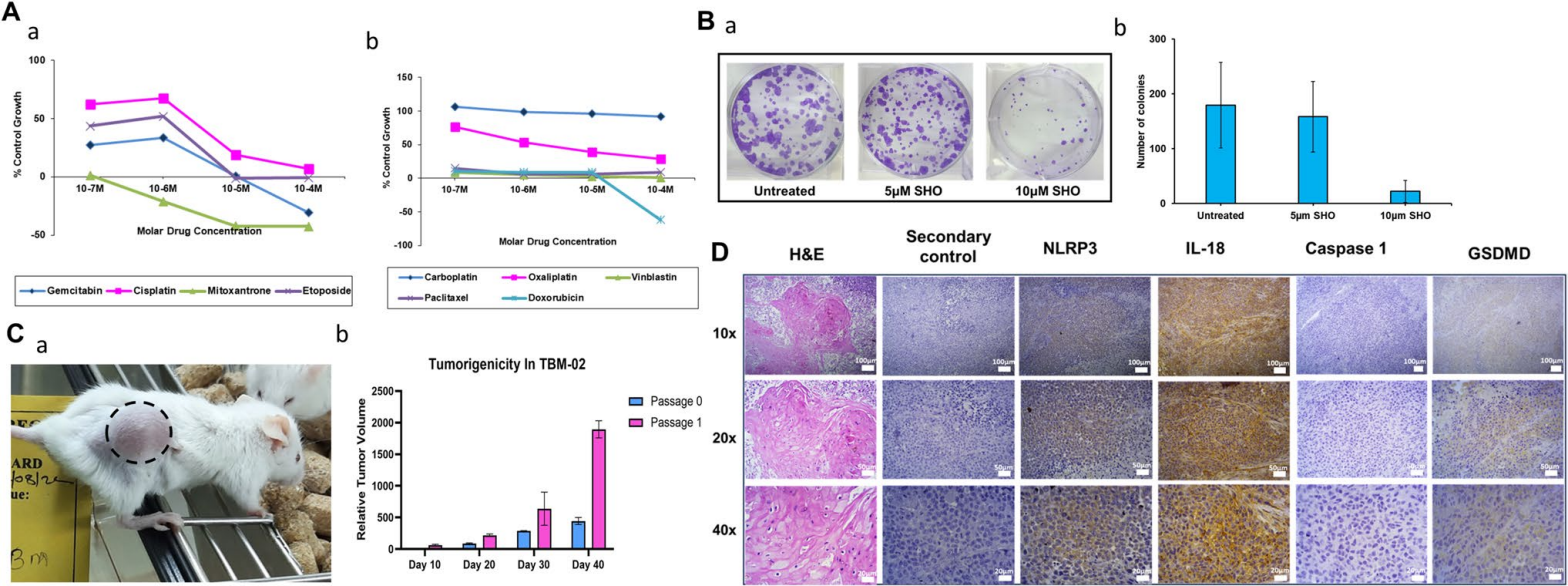
Functional potential of TBM-02. The line graphs (A) demonstrate chemosensitivity profile of TBM-02 for standard chemotherapeutic drugs. The clonogenicity of TBM-02 is shown by colony formation assay in presence and absence of phytochemical 6-Shogaol (5 µM and 10 μM), the plates were stained with crystal violet dye. The reduction in number after treatment is depicted in bar graph (subpanel Bb). Tumorigenic potential of TBM-02 was tested by subcutaneous injection of cells in immunodeficient NOD-SCID mice. The tumor growth was monitored on regular intervals, and line graph (subpanel Ca) depicts the growth curve of TBM-02 in vivo. The representative xenograft of TBM-02 is shown in subpanel Cb. The xenograft tumor was processed to make paraffin blocks, and the sections were stained by immunohistochemistry for NLRP3 Inflammasome pathway markers (NLRP3, IL-18, Caspase-1, GSDMD, and AIM-2). The sections having tumor area stained for various markers are shown in panel D. The sections stained with only secondary antibody served as staining control. H&E-stained section was used to map the tumor area

Drug sensitivity assay (Fig. 3A) demonstrated that TBM-02 was sensitive to vinblastine (IC_50_−0.032 μM), paclitaxel (IC_50_−0.04 μM), doxorubicin (IC_50_−0.1085 μM), gemcitabine (IC_50_−0.36 μM), cisplatin (IC_50_−8.808 μM), mitoxantrone (IC_50_−0.043 μM), and etoposide (IC_50_−0.787 μM). TBM-02 cells were sensitive to phytochemical 6-shogaol (IC_50_−20 μM) (Supplementary Fig. S3) also. TBM-02 cells were able to form colonies (Fig. 3B-a, B-b) at a seeding density as low as 1500 cells. Further colony formation potential of TBM-02 showed a dose-dependent decrease in presence of 6-Shogaol.

TBM-02 when injected subcutaneously into the flank of NOD-SCID mice was able to form tumors as early as 20 days of injection (Fig. 3C-a, C-b). The first serial passage was done at day 40, and subsequent serial passages were at intervals of 25–30 days. The tumors were passaged up to 5 passages and stored in liquid nitrogen for future use. The histopathology by H&E staining showed that TBM-02 formed poorly differentiated oral squamous cell carcinoma (Fig. 3D). The markers of chronic inflammation NLRP3, IL-18, and GSDMD stained positive in the xenograft tumors.

### Identification of pathways associated with oral cancer by transcriptome analysis

**Fig. 4:**
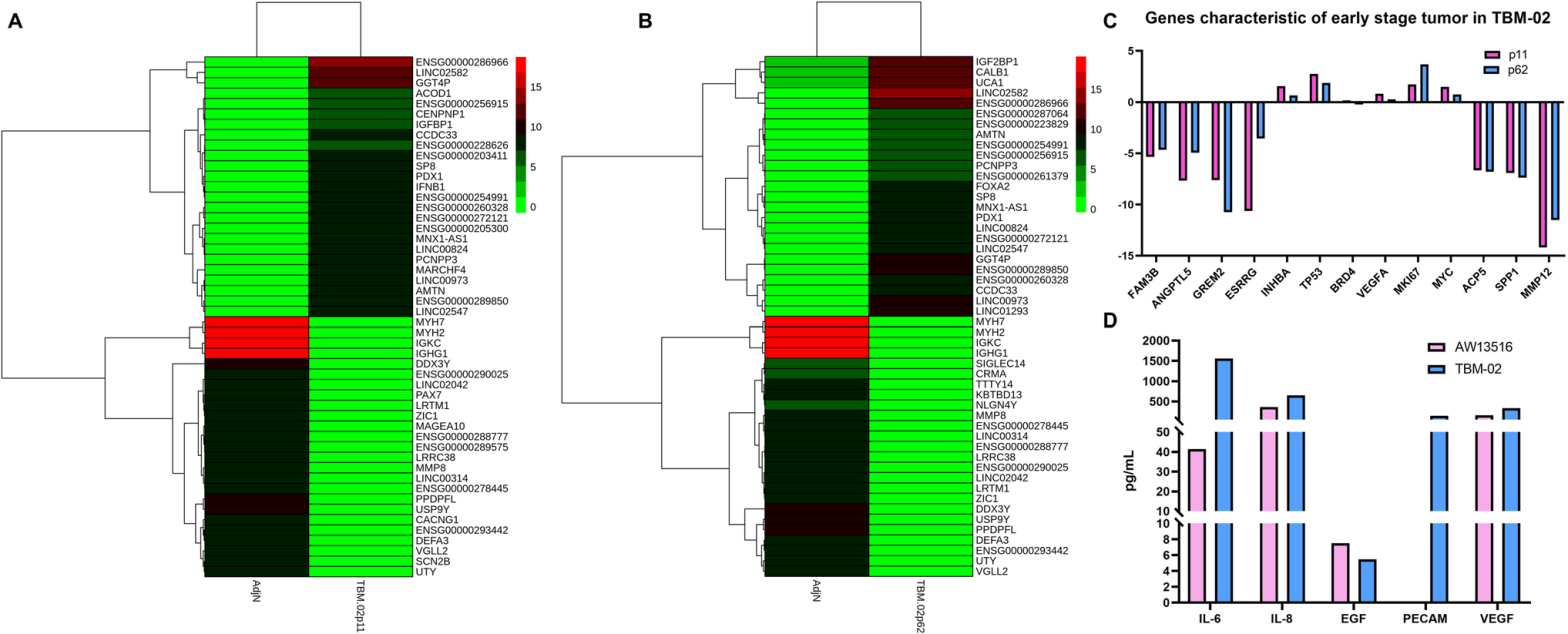
Transcriptomic and proteomic analysis of TBM-02 cell line. Heatmaps demonstrate the differentially expressed genes of TBM-02 cell line at early passage p11 (**A**) and late passage p62 (**B**) in comparison to normal buccal mucosa (SRA database SRX19125628 and SRX19125629). **C** Bar graph demonstrates selected differentially expressed genes in TBM-02 at p11, and p62 FAM3B to INHBA were selected from our own patient data of genes expressed differentially in early stage tumor versus corresponding adjacent normal. TP53 to MYC were selected from literature as markers characteristic of early stage oral tumor. Genes ACP5, SPP1, and MMP12 selected from our own patient data of genes expressed differentially in late-stage tumor versus corresponding adjacent normal. **D** Secreted cytokines and growth factors in TBM-02 (p62) and AW13516 cell line supernatant

To delineate the key pathways involved in oral cancer, the transcriptome analysis TBM-02 at early passage (p11) and late passage (p62) was compared with that of normal buccal mucosa (SRA database SRX19125628 and SRX19125629). Out of ~ 50 million reads, DGE analysis showed that 7456 genes were downregulated and 5331 genes were upregulated at early passage (p11), whereas 7086 genes were downregulated and 4068 genes were upregulated at late passage (p62) in comparison to normal buccal mucosa. Heatmaps show variation in transcriptome profiles of TBM-02 at p11 (Fig. 4A) and p62 (Fig. 4B). Gene ontology (GO) enrichment analysis shows that Wnt signaling pathway, DNA replication, and double-strand break repair genes are upregulated in TBM-02 in comparison to the normal buccal mucosa (Supplementary Fig. S4-A and S4-B). To check the major pathways involved in transformation of cells, we also performed Kyoto Encyclopedia for Genes and Genomes (KEGG) pathway enrichment analysis, which revealed that MAPK signaling, cell cycle, Rap 1 signaling, Ras signaling, NF-κB signaling, cellular senescence, Hippo signaling, TGF-β signaling, p53 signaling, and PI3K-AKT pathways were dysregulated in the cell line in both early (Supplementary Fig. S4-C) and late passages (Supplementary Fig. S4D) in comparison to the normal buccal mucosa.

To find early stage oral tumor specific markers in TBM-02, we selected few genes from our own study on oral cancer buccal mucosa patients. These genes were differentially expressed in early stage tumor versus corresponding adjacent normal (data not shown). Expression pattern of genes observed in in oral buccal mucosa cancer patients (upregulated: INHBA, downregulated: FAM3B, ANGPTL5, GREM2 and ESRRG) corroborated with that observed in TBM-02 at both p11 and p62 passages (Fig. 4C and Supplementary Table S4). Further, we selected genes (ACP5, SPP1, and MMP12) from our study on late-stage oral cancer buccal mucosa patients. These genes were differentially upregulated in late-stage tumor compared to corresponding adjacent normal (data not shown). In contrast, these genes were downregulated in TBM-02 which represents cell line from early-stage tumor (Fig. 4C and Supplementary Table S4). Next, we selected few genes from literature which were characteristic of early stage oral tumor. In corroboration with published literature, TBM-02 demonstrated upregulated TP53, MYC, Ki-67, VEGFA, and BRD4 (Fig. 4C). At proteomic level, TBM-02 cell line supernatant demonstrated distinct levels of cytokines IL-6 and IL-8, and growth factors EGF, PECAM-1 and VEGF in comparison to AW13516 which is a tongue cancer cell line (Fig. 4D).

### TBM-02 as a pre-clinical model for therapeutic targeting of NLRP3

**Fig. 5:**
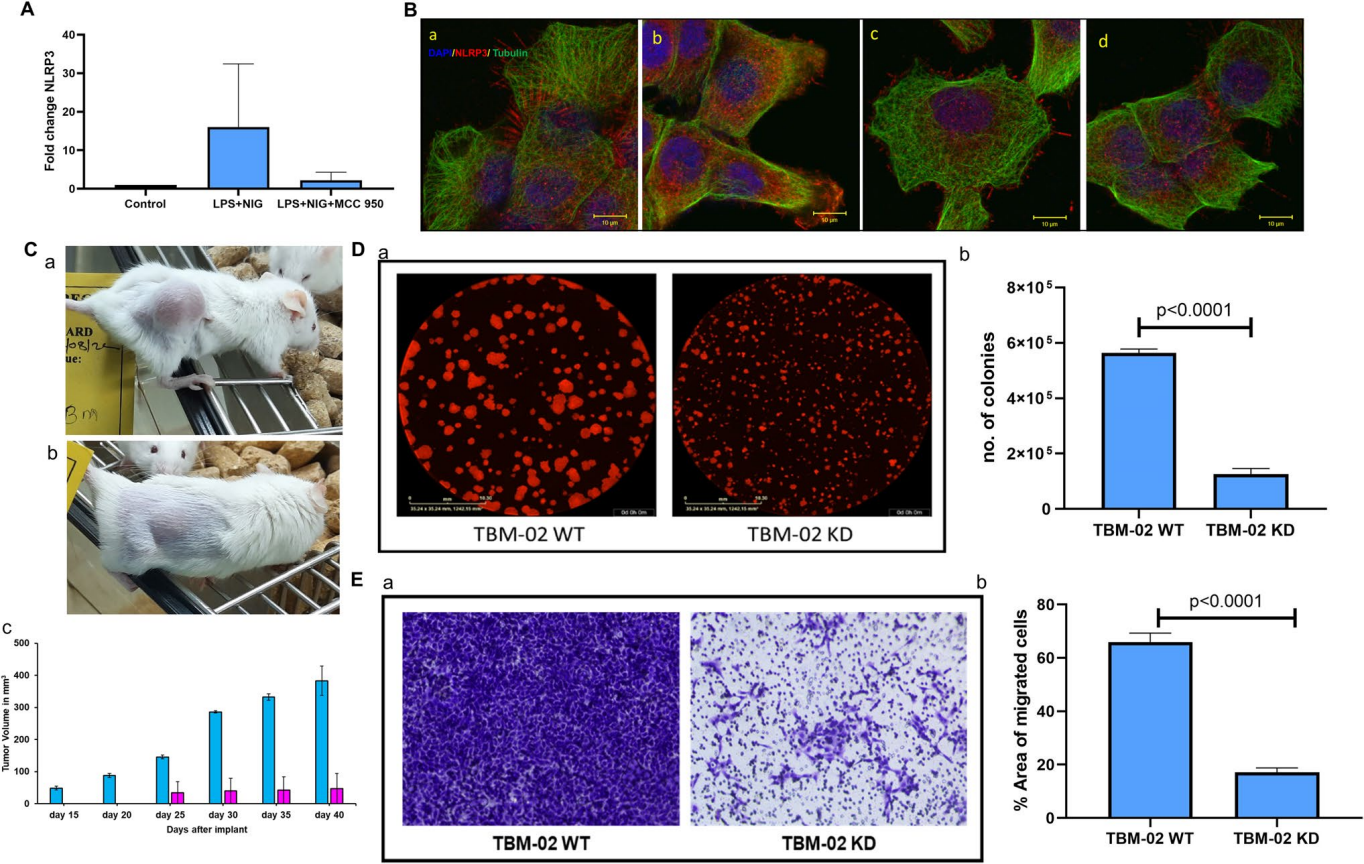
Demonstration of NLRP3 as therapeutic target using TBM-02 cell line. TBM-02 cells were activated for inflammasome pathway with LPS (500 ng/ml) and Nigericin (5 µM) and inhibited using standard NLRP3 inhibitor MCC950 (5 µM) and 6-Shogaol (5 µM).Transcript expression (**A**) and protein expression (**B**) of NLRP3 was demonstrated by Real Time-PCR and immunofluorescence coupled with confocal microscopy, respectively. The images shown in panel B reveal spatial distribution of NLRP3 in TBM-02 in presence/absence of activation/inhibition of inflammasome pathway. NLRP3 expression is seen in red color, whereas tubulin is seen in green color. The untreated TBM-02 exhibit even distribution of NLRP3 and organized tubulin architecture (**Ba**). Upon inflammasome activation, expression of NLRP3 increased and TBM-02 showed co-localization of NLRP3 with tubulin (crystalline nature) in more organized architecture (**Bb**). The inhibitor MCC950 (**Bc**) and 6-Shogaol (**Bd**) demonstrated significant inhibition of inflammasome pathway leading to reduction of NLRP3 expression and tubulin depolymerization (amorphous nature). To show the association of NLRP3 with tumorigenicity, the gene was knocked-down by lentivirus-mediated sh-RNA. The NOD-SCID mice were injected subcutaneously and the representative mouse mouse for wild type TBM-02 WT (**Ca**) and Knock-down TBM-02 KD (**Cb**) group is shown. The mice were followed for tumor volume, measured at regular intervals. The comparative growth curve indicated that the knockdown of NLRP3 led to significant reduction and delay in tumor growth (subpanel **Cc**). The knocking down of NLRP3 also led to significant reduction in clonogenic (**D**) and migration (**E**) potential of TBM-02 as seen by colony formation assay. The reduction in number of colonies of TBM-02 WT and TBM-02 KD cells, after staining culture plates with Sulphorhodamine-B (SRB) and observing under confocal microscope, is depicted in bar graph (subpanel **Db**). For trans-well migration assay, plates were stained with crystal violet and area of cells migrated was calculated and plotted in the bar graph (subpanel **Eb**)

At genomic level, NLRP3 transcript expression was found to be increased in inflammation activation model of TBM-02 over untreated cells. This got further diminished in presence of NLRP3 specific inhibitor MCC 950. (Fig. 5A). The assembly of NLRP3 inflammasome was also checked by immunofluorescence for assembly of NLRP3 multimer where we observed that on activation, there is an aggregation of NLRP3 and inhibition on treatment with MCC950 and phytochemical 6-Shogaol (Fig. 5B). Then we generated stable knockdown of NLRP3 cell line in TBM-02 and checked the effect of NLRP3 knockdown by tumorigenesis, colony formation, and trans-well migration assay. The NLRP3 knockdown cells show significantly low proliferation as seen by colony formation assay (Fig. 5D-a, D-b). Tumorigenesis in NOD-SCID mice is also compromised in knockdown cells in comparison to wild type cells (Fig. 5C-a, C-b, C–c). Migration assay also shows that NLRP3 knockdown cells have a very low migratory potential across the trans-well membrane (Fig. 5E-a, E-b).

## Discussion

Asia has the highest number of incidences, mortality, and prevalence of OSCC in the world [26]. Etiology can be attributed to socioeconomic habit of using smokeless tobacco which is prevalent in South and East Asia and is associated with South Asian ethnicity all over the world [27]. The efforts were focused on establishing oral cancer cell lines which could be used as a model for screening potential drugs for oral cancer therapeutics. However, the oral tumor origin was Caucasian population [28] which may not be appropriate to screen the efficacy of potential drugs to be designed for Indian population. In this context, we generated and established cell line from early stage oral cancer of buccal mucosa from patient of Indian origin.

The oral cancer buccal mucosa cell lines reported previously have validated the epithelial origin of oral cancer cell lines by staining for cytokeratin 8 and 14 [29] or EpCAM [30]. In alignment with these observations, TBM-02 also expressed high levels of EpCAM, cytokeratin 8, and cytokeratin 14. It was interesting to note that the genes we had obtained from early stage buccal mucosa oral cancer patient tumor clinical samples (data not shown) as differentially regulated genes over their corresponding adjacent normal were found to be distinctly expressed in both early and late passages of TBM-02. To evaluate more of early stage tumor characteristic genes, the genes obtained from late-stage tumors (data not shown) were found to be oppositely regulated in TBM-02. The early stage buccal mucosa tumor-associated genes viz. TP53 [31], BRD4 [32], Myc [33], and Ki67 [34] were found to be distinctly upregulated in TBM-02.

There are reports that exposure to carcinogenic agents in smokeless tobacco leads to chromosomal instability and aneuploidy advancing to field cancerization [35]. Further few others [7, 8] also reported loss of Y allele and aneuploid DNA content in the tongue cancer cell lines established from advanced stage tongue and buccal mucosa cancer patients. In our study, TBM-02 exhibited aneuploidy, abnormal chromosomal content consisting of 69 chromosomes with insertions as well as deletions of few chromosomes, especially loss of Y chromosome. This was also corroborated by STR analysis. Loss of Y can be attributed to repeated passaging of cells and has also been reported by other groups who have established cell line from patients of Indian origin [8].

TBM-02 revealed high clonogenicity and enhanced migration in trans-well migration assay demonstrating metastatic potential. Clonogenicity and migration property being the hallmark of transformed cells [36], these properties of the cell line allow screening of potential drugs against metastasis. Similarly, others [37] have reported that oral cancer cell lines demonstrate migratory capability due to epithelial to mesenchymal transformation, associated with ultrastructure and TP53 mutation status in the genome.

Tumor forming ability in mice model is another peculiar characteristic of established cell line [38]. In our study, TBM-02 could give rise to CDX which could be serially maintained up to 5 passages. Other groups from India have also reported similar CDX formation ability along with PET-SCAN and lymph node metastasis [7, 8]; however, they did not address inflammation in their model. As against, we demonstrated the association of chronic inflammation with TBM-02 xenograft in terms of upregulated expression of NLRP3 inflammasome pathway markers viz. NLRP3, IL-18, and Caspase 1. Co-clinical trials using mice CDX or patient-derived xenograft (PDX) have been useful to stratify human clinical trials and guide in selecting appropriate chemotherapeutic agents [39, 40]. Co-clinical trials include conducting of pre-clinical studies and clinical trials simultaneously, thus allowing real-time data integration to accurately stratify and tailor therapy for personalized treatment.

Others and our own previous study have shown oral cancer cell line can be used as a screening model for evaluating efficacy of chemotherapeutic drugs [41, 42]. TBM-02 also demonstrated variable efficacy to an array of standard chemotherapeutic drugs implicating its potential use as a pre-clinical screening model. The cells are sensitive to standard chemotherapeutic drugs cisplatin and paclitaxel which are used in treatment regime of oral cancer [43].

Furthermore, using LPS and Nigericin stimulation, we also established inflammasome activation model which can be used to screen potential anti-cancer drugs and inhibitors that act through regulation of inflammation and inflammasome pathway [44]. Similarly, others have reported using classical THP-1 cell lines characterization and development of novel inhibitors Nitroxoline [45] and Nicardipine [46] acting through NLRP3-dependent pyroptosis.

TBM-02 can also be used for validating therapeutic targets. In our study, we demonstrated NLRP3 as therapeutic target associated with initiation and promotion. By knocking down NLRP3 gene in TBM-02, we found that there is a decrease in proliferation and migration of cancer cells in comparison to wild-type cells. Others have reported that downregulation of NLRP3 inhibited proliferation, migration, and invasion of OSCC in in vivo model. They also correlated the NLRP3 expression level with tumor size and metastatic status [11]. Another study by Ref. [47] demonstrated that NLRP3 pathway molecules were overexpressed in HNSCC tissue and *Tgfbr1/Pten* 2cKO model. They correlated the levels of CSC (cancer stem cell) markers with NLRP3 inflammasome and concluded that NLRP3 promotes CSC formation and might play a role in regulation of CSCs in HNSCC. Chen et al. [48] were able to show that inhibition of NLRP3 pathway by MCC-950 reduced the IL-1β secretion and delayed tumorigenesis in spontaneous de novo HNSCC model mice. NLRP3 inhibition also led to reduced infiltration of immunosuppressive immune cells such as Tregs, MDSCs, and TAMs and led to immunomodulation by decreasing PD-1 and Tim-3 exhausted T-cells and increasing TILs. In this context, TBM-02 can also be used as tool to identify and establish NLRP3 as well as other therapeutic targets of oral cancer.

Established cancer cell lines provide a platform to delineate mutations and variations associated with transformation of cells which can be assessed through high-throughput techniques like whole exome sequencing, transcriptome analysis, whole genome sequencing, ATAC sequencing, spatial transcriptomics, and single cell sequencing [49]. Here, in our study, using transcriptome analysis of TBM-02 in comparison with normal buccal mucosa, we found distinct signature metabolic and molecular pathways involved in cancer development.

## Conclusion

Our current study has described successful generation, characterization, and establishment of early stage oral cancer cell line which can be explored and developed further as pre-clinical tool for screening potential oral cancer drugs, tool for gaining insights into underlying disease progression mechanism, and identify therapeutic targets for early-stage oral cancer therapeutics. Thus, we have reported a novel oral cancer cell line of Indian origin which provides cost effective, easy to use model with unlimited supply of material, and bypassing ethical concern for developing new affordable drugs for oral cancer management.

## Supplementary Information

Below is the link to the electronic supplementary material.

Supplementary file 1 (DOCX 26 KB)

Supplementary file 2 (DOCX 701 KB)

Supplementary file 3 (DOCX 23 KB)

## Data Availability

The datasets generated will be made available on request. This invention is successfully published by Indian Patent Office. Dr. Jyoti Kode, Ms. Akhila George, Mrs. Trupti Pradhan, Dr. Poonam Gera, Dr. Kumar Prabhash, Dr. Sudhir Nair, Dr. Nirmal Kumar K., Mr. Subrata Sen, Mr. Madan Barkume. An establishment and characterization of an oral cancer cell line model of Indian origin ‘TBM-02’ and applications thereof. Indian Patent Application # 202421061738 published on 18th July, 2025.

## References

[1] Nocini R, Lippi G, Mattiuzzi C. The worldwide burden of smoking-related oral cancer deaths. Clin Exp Dent Res. 2020;6:161–4. 10.1002/cre2.265. 32250564 10.1002/cre2.265 PMC7133730

[2] Lokhande M, Kannusamy S, Oak A, Cheulkar S, Chavan S, Mishra V, et al. A hospital-based study of survival in oral cancer patients of Tata Memorial Hospital, Mumbai. Ecancermedicalscience. 2024;18:1–12. 10.3332/ecancer.2024.1669. 10.3332/ecancer.2024.1669 PMC10911663 38439812

[3] Misra S, Chaturvedi A, Misra NC. Management of gingivobuccal complex cancer. Ann R Coll Surg Engl. 2008;90:546–53. 10.1308/003588408X301136. 18701010 10.1308/003588408X301136 PMC2728300

[4] Gupta B, Ariyawardana A, Johnson NW. Oral cancer in India continues in epidemic proportions: evidence base and policy initiatives. Int Dent J Engl. 2013;63:12–25. 10.1111/j.1875-595x.2012.00131.x. 10.1111/j.1875-595x.2012.00131.x PMC9374955 23410017

[5] Borse V, Konwar AN, Buragohain P. Oral cancer diagnosis and perspectives in India. Sens Int. 2020;1:100046. 10.1016/j.sintl.2020.100046. 34766046 10.1016/j.sintl.2020.100046 PMC7515567

[6] Kaur G, Dufour JM. Cell lines. Spermatogenesis. 2012;2:1–5. 10.4161/spmg.19885. 22553484 10.4161/spmg.19885 PMC3341241

[7] Joshi P, Bane S, Chaturvedi P, Gera P, Waghmare SK. Establishment and characterization of patient-derived tongue squamous cell carcinoma cell lines. Hum Cell. 2025;38:1–11. 10.1007/s13577-025-01231-w. 10.1007/s13577-025-01231-w PMC12092557 40394426

[8] Pansare K, Gardi N, Kamat S, Dange P, Previn R, Gera P, et al. Establishment and genomic characterization of gingivobuccal carcinoma cell lines with smokeless tobacco associated genetic alterations and oncogenic PIK3CA mutation. Sci Rep. 2019;9:1–10. 10.1038/s41598-019-44143-0. 31164688 10.1038/s41598-019-44143-0 PMC6547758

[9] Sharma BR, Kanneganti TD. NLRP3 inflammasome in cancer and metabolic diseases. Nat Immunol. 2021;22:550–9. 10.1038/s41590-021-00886-5. 33707781 10.1038/s41590-021-00886-5 PMC8132572

[10] Bauernfeind F, Horvath G, Stutz A, Alnemri ES, Speert D, Fernandes-alnemri T, et al. NF-kB activating pattern recognition and cytokine receptors license NLRP3 inflammasome activation by regulating NLRP3 expression. J Immunol. 2010;183:787–91. 10.4049/jimmunol.0901363.NF-kB. 10.4049/jimmunol.0901363 PMC2824855 19570822

[11] Wang H, Luo Q, Feng X, Zhang R, Li J, Chen F. NLRP3 promotes tumor growth and metastasis in human oral squamous cell carcinoma. BMC Cancer. 2018;18:1–10. 10.1186/s12885-018-4403-9. 29716544 10.1186/s12885-018-4403-9 PMC5930757

[12] Xu Z, Wang H, Qin Z, Zhao F, Zhou L, Xu L, et al. NLRP3 inflammasome promoted the malignant progression of prostate cancer via the activation of caspase-1. Cell Death Discov. 2021;7:1–9. 10.1038/s41420-021-00766-9. 10.1038/s41420-021-00766-9 PMC8688424 34930938

[13] Tatake RJ, Rajaram N, Damle RN, Balsara B, Bhisey AN, Gangal SG. Establishment and characterization of four new squamous cell carcinoma cell lines derived from oral tumors. J Cancer Res Clin Oncol. 1990;116:179–86. 10.1007/BF01612674. 1691185 10.1007/BF01612674 PMC12200902

[14] Lilly E, Almeida JL, Korch CT. Authentication of human and mouse cell lines by short tandem repeat (STR) DNA genotype analysis [internet]; 2004. https://www.ncbi.nlm.nih.gov/books/. 23805434

[15] Shi S-R, Key ME, Kalra KL. Antigen retrieval in formalin-fixed, paraffin-embedded tissues: an enhancement method for immunohistochemical staining based on microwave oven heating of tissue sections; 1991. 10.1177/39.6.1709656 1709656

[16] Yong-kie Wong D, Chang K, Chen C, Che-shoa Chang R. Characterization of two new cell lines derived from oral cavity human squamous cell carcinomas-OC7 and OC2. 10.1016/0278-2391(90)90436-6 2156034

[17] Lin S-C, Liu C-J, Chiu C-P, Chang S-M, Lu S-Y, Chen Y-J. Establishment of OC3 oral carcinoma cell line and identi^®^cation of NF-kB activation responses to areca nut extract [Internet]. http://research.marsh. 10.1111/j.1600-0714.2004.00034.x 14720193

[18] Ganjibakhsh M, Monshizadeh R, Nasimian A, Aminishakib P, Farzaneh P, Tavakoli Shiraji S, et al. Anti-angiogenic efficacy of aflibercept and bevacizumab in primary oral squamous cell carcinoma cells. J Oral Pathol Med. 2018;47:575–82. 10.1111/jop.12717. 29672933 10.1111/jop.12717

[19] Ganjibakhsh M, Aminishakib P, Farzaneh P, Karimi A, Abolhassan S, Fazeli S, et al. of Head and neck; primary cell culture [internet]. Tehran University of Medical Sciences. 2017. www.jdt.tums.ac.ir.

[20] Zaki Hidayatullah Fadlullah M, Kim-Ni Chiang I, Dionne KR, San Yee P, Phei Gan C, Kit Sam K, et al. Genetically-defined novel oral squamous cell carcinoma cell lines for the development of molecular therapies [Internet]. Oncotarget. www.impactjournals.com/oncotarget/. 10.18632/oncotarget.8533 PMC5053689 27050151

[21] White JS, Weissfeld JL, Ragin CCR, Rossie KM, Martin CL, Shuster M, et al. The influence of clinical and demographic risk factors on the establishment of head and neck squamous cell carcinoma cell lines. Oral Oncol. 2007;43:701–12. 10.1016/j.oraloncology.2006.09.001. 17112776 10.1016/j.oraloncology.2006.09.001 PMC2025692

[22] Russell SM, Lechner MG, Gong L, Megiel C, Liebertz DJ, Masood R, et al. USC-HN2, a new model cell line for recurrent oral cavity squamous cell carcinoma with immunosuppressive characteristics. Oral Oncol. 2011;47:810–7. 10.1016/j.oraloncology.2011.05.015. 21719345 10.1016/j.oraloncology.2011.05.015 PMC3164740

[23] Lepikhova T, Karhemo PR, Louhimo R, Yadav B, Murumagi A, Kulesskiy E, et al. Drug-sensitivity screening and genomic characterization of 45 hpV-negative head and neck carcinoma cell lines for novel biomarkers of drug efficacy. Mol Cancer Ther. 2018;17:2060–71. 10.1158/1535-7163.MCT-17-0733. 29970484 10.1158/1535-7163.MCT-17-0733

[24] Van Zeeburg HJT, Snijders PJF, Pals G, Hermsen MAJA, Rooimans MA, Bagby G, et al. Generation and molecular characterization of head and neck squamous cell lines of Fanconi anemia patients [Internet]; 2005. www.aacrjournals.org. 10.1158/0008-5472.CAN-04-3665 15735012

[25] Lee EJ, Kim J, Lee SA, Kim E-J, Chun Y-C, Ryu MH, et al. Characterization of newly established oral cancer cell lines derived from six squamous cell carcinoma and two mucoepidermoid carcinoma cells. Exp Mol Med. 2005;37:379–90. 10.1038/emm.2005.48. 16264262 10.1038/emm.2005.48

[26] Bray F, Laversanne M, Sung H, Ferlay J, Siegel RL, Soerjomataram I, et al. Global cancer statistics 2022: GLOBOCAN estimates of incidence and mortality worldwide for 36 cancers in 185 countries. CA Cancer J Clin. 2024;74:229–63. 10.3322/caac.21834. 38572751 10.3322/caac.21834

[27] Scully C, Bedi R. Ethnicity and oral cancer. Lancet Oncol. 2000;1:37–42. 10.1016/S1470-2045(00)00008-5. 11905687 10.1016/S1470-2045(00)00008-5

[28] Zaaijer S, Capes-Davis A. Ancestry matters: building inclusivity into preclinical study design. Cell. 2021;184:2525–31. 10.1016/j.cell.2021.03.041. 33989545 10.1016/j.cell.2021.03.041

[29] Gawas NP, Navarange SS, Chovatiya GL, Chaturvedi P, Waghmare SK. Establishment and characterization of novel human oral squamous cell carcinoma cell lines from advanced-stage tumors of buccal mucosa. Oncol Rep. 2019;41:2289–98. 10.3892/or.2019.7003. 30816493 10.3892/or.2019.7003

[30] Imrich S, Hachmeister M, Gires O. EpCAM and its potential role in tumor-initiating cells. Cell Adh Migr. 2012;6:30–8. 10.4161/cam.18953. 22647938 10.4161/cam.18953 PMC3364135

[31] Liu Y, Su Z, Tavana O, Gu W. Understanding the complexity of p53 in a new era of tumor suppression. Cancer Cell. 2024. 10.1016/j.ccell.2024.04.009. 38729160 10.1016/j.ccell.2024.04.009 PMC11190820

[32] Hu J, Pan D, Li G, Chen K, Hu X. Regulation of programmed cell death by Brd4. Cell Death Dis. 2022. 10.1038/s41419-022-05505-1. 36539410 10.1038/s41419-022-05505-1 PMC9767942

[33] Whitfield JR, Soucek L. MYC in cancer: from undruggable target to clinical trials. Nat Rev Drug Discov. 2025. 10.1038/s41573-025-01143-2. 39972241 10.1038/s41573-025-01143-2 PMC7619205

[34] Li LT, Jiang G, Chen Q, Zheng JN. Ki67 is a promising molecular target in the diagnosis of cancer (Review). Mol Med Rep. 2015. 10.3892/mmr.2014.2914. 25384676 10.3892/mmr.2014.2914

[35] Giaretti W, Monteghirfo S, Pentenero M, Gandolfo S, Malacarne D, Castagnola P. Chromosomal instability, DNA index, dysplasia, and subsite in oral premalignancy as intermediate endpoints of risk of cancer. Cancer Epidemiol Biomarkers Prev. 2013;22:1133–41. 10.1158/1055-9965.EPI-13-0147. 23629518 10.1158/1055-9965.EPI-13-0147

[36] Hanahan D, Weinberg RA. Hallmarks of cancer: the next generation. Cell. 2011. 10.1016/j.cell.2011.02.013. 21376230 10.1016/j.cell.2011.02.013

[37] Navarange SS, Bane SM, Mehta D, Shah S, Gupta S, Waghmare SK. Epithelial-to-mesenchymal transition status correlated with ultrastructural features, and TP53 mutation in patient-derived oral cancer cell lines. Mol Biol Rep. 2023;50:8469–81. 10.1007/s11033-023-08720-x. 37639153 10.1007/s11033-023-08720-x

[38] Gillet JP, Varma S, Gottesman MM. The clinical relevance of cancer cell lines. J Natl Cancer Inst. 2013. 10.1093/jnci/djt007. 23434901 10.1093/jnci/djt007 PMC3691946

[39] Balasubramanian B, Venkatraman S, Myint KZ, Janvilisri T, Wongprasert K, Kumkate S, et al. Co-clinical trials: an innovative drug development platform for cholangiocarcinoma. Pharmaceuticals. 2021. 10.3390/ph14010051. 33440754 10.3390/ph14010051 PMC7826774

[40] Kim HR, Kang HN, Yun MR, Ju KY, Choi JW, Jung DM, et al. Mouse–human co-clinical trials demonstrate superior anti-tumour effects of buparlisib (BKM120) and cetuximab combination in squamous cell carcinoma of head and neck. Br J Cancer. 2020;123:1720–9. 10.1038/s41416-020-01074-2. 32963347 10.1038/s41416-020-01074-2 PMC7722843

[41] Patil TT, Kowtal PK, Nikam A, Barkume MS, Patil A, Kane SV, et al. Establishment of a tongue squamous cell carcinoma cell line from Indian gutka chewer. J Oral Oncol. 2014;2014:1–9. 10.1155/2014/286013.

[42] Kode J, Kovvuri J, Nagaraju B, Jadhav S, Barkume M, Sen S, et al. Synthesis, biological evaluation, and molecular docking analysis of phenstatin based indole linked chalcones as anticancer agents and tubulin polymerization inhibitors. Bioorg Chem. 2020. 10.1016/j.bioorg.2020.104447. 33207276 10.1016/j.bioorg.2020.104447

[43] Chaturvedi P, Prabhash K, Babu G, Kuriakose M, Birur P, Anand A, et al. Indian clinical practice consensus guidelines for the management of oral cavity cancer. Indian J Cancer. 2020;57:S6-8. 10.4103/0019-509X.278975. 32167064 10.4103/0019-509X.278975

[44] Balahura Stămat LR, Dinescu S. Inhibition of NLRP3 inflammasome contributes to paclitaxel efficacy in triple negative breast cancer treatment. Sci Rep. 2024. 10.1038/s41598-024-75805-3. 39433537 10.1038/s41598-024-75805-3 PMC11494052

[45] Maeder C, Baumann R, Gaul S, Fikenzer S, Schaefer M, Kalwa H, et al. Nitroxoline is a novel inhibitor of NLRP3-dependent pyroptosis. Cell Death Discov. 2025. 10.1038/s41420-025-02699-z. 40835597 10.1038/s41420-025-02699-z PMC12368067

[46] Chang Y, Jean W, Lu CW. Nicardipine inhibits priming of the NLRP3 inflammasome via suppressing LPS-induced TLR4 expression. Inflammation. 2020;43:1375–86. 32239395 10.1007/s10753-020-01215-y

[47] Huang CF, Chen L, Li YC, Wu L, Yu GT, Zhang WF, et al. NLRP3 inflammasome activation promotes inflammation-induced carcinogenesis in head and neck squamous cell carcinoma. J Exp Clin Cancer Res. 2017;36:1–13. 10.1186/s13046-017-0589-y. 28865486 10.1186/s13046-017-0589-y PMC5581464

[48] Chen L, Huang CF, Li YC, Deng WW, Mao L, Wu L, et al. Blockage of the NLRP3 inflammasome by MCC950 improves anti-tumor immune responses in head and neck squamous cell carcinoma. Cell Mol Life Sci. 2018;75:2045–58. 10.1007/s00018-017-2720-9. 29184980 10.1007/s00018-017-2720-9 PMC11105265

[49] Talsania K, Shen WT, Chen X, Jaeger E, Li Z, Chen Z, et al. Structural variant analysis of a cancer reference cell line sample using multiple sequencing technologies. Genome Biol. 2022. 10.1186/s13059-022-02816-6. 36514120 10.1186/s13059-022-02816-6 PMC9746098

