# Supplementary material for: Generation and characterization of early stage oral cancer cell line of buccal mucosa of Indian origin
Source: Hum Cell. 2025 Dec 18;39(1):21. doi: 10.1007/s13577-025-01332-6 (PMC12714774; doi:10.1007/s13577-025-01332-6)
Supplement: Supplementary file 1 — (DOCX 26 KB) [file 13577_2025_1332_MOESM1_ESM.docx]

**Generation and characterization of Early-stage Oral Cancer cell line of Buccal mucosa of Indian Origin**

Akhila George^1,2^, Sudhir Nair ^2,3,4^, Kumar Prabhash^2,4,5,6^, Sayujata Thakur^1^, Poonam Gera^7^, Arjun Singh^2,3,4^, Pankaj Chaturvedi^2,3,4^, Swapnil Rane^2,4,8^, Trupti Pradhan^1^, Subrata Sen^9^, Madan Barkume^9^, Dhanlaxmi Shetty^2^,^10^, Kruti Chaubal^10^, Arpita Ghosh^11^, Sanjeev Kamte^11^, Jyoti Kode^1,2,9*^

**2.2**. **Buffers:**

1. **Phosphate buffered saline**:

**Solution A**: 0.2M NaH_2_PO_4_.2H_2_O: 5.616-gram NaH_2_PO_4_.2H_2_O as dissolved in 180 mL deionized water.

**Solution B**: 0.2M Na_2_HPO4.2H_2_O: 32.03-gram 0.2M Na_2_HPO4.2H_2_O was dissolved in 900 mL deionized water.

160 ml solution and 840 ml solution B was mixed properly and pH was adjusted to 7.5. To this, 170 grams NaCl was added, dissolved properly and volume was made upto 2000ml. The solution was stored at 4˚C

1. **FACS buffer**: 2mL Fetal Bovine Serum ( FBS) and 0.02grams Sodium Azide (NaN_3_) was added to 98 ml of 1X PBS
2. **Dilution buffer**: 5grams and 0.1 ml of NP-40 of bovine serum albumin was added to 100 mL 1X PBS
3. **Wash buffer**: 0.1 ml of NP-40 of bovine serum albumin was added to 100 mL 1X PBS

**2.5. Brief Protocol of mycoplasma detection kit**:

The kit used was Mycoplasma Detection Kit-Quick test. The principle of the assay is that mycoplasma metabolises the media components to produce metabolites which specific to it. The kit detects these specific metabolites to detect mycoplasma load in the culture. The unused media is used as negative control. 40μl reaction buffer A is added to the reaction wells with 10μl positive control, negative control, and test samples and incubated for 5 minutes. The test samples taken is cell culture supernatant obtained after 36-48 hours continuous culture. Then 40ul Reaction buffer B is added to all the wells and incubated for 4 mins. Next 5 µl stop solution is added and observed for colour change for about 30 minutes.

- - 1. **Karyotyping and ploidy analysis**

The chromosome profiling of TBM-02 was done by karyotyping at passage 5 and 67 to assess tumor cell heterogeneity and chromatin stability upon passaging. 1 X 10^6^ cells were cultured in 75 cm^2^ flask and incubated at 37˚C for 24 hours. The cells were washed with PBS (pH – 7.4) and treated with colchicine in complete IMDM supplemented with 10% FBS for 17 hours to arrest the cells at metaphase. Then the cells were trypsinized and further harvested using pre-warmed (37˚C)0.075M KCL for 40 mins followed by three washes of chilled Carnoy’s fixative (3:1; methanol: glacial acetic acid). The cells obtained were then dropped on glass slides and banded by GTG method. The metaphases were captured and analysed using ASI GenASIs software. Further confirmations of chromosomal aberrations were done by metaphase FISH using WCP (whole chromosome paints) probes using standard FISH protocol.

- - 1. **Brief protocol of Transmission electron microscopy:**

For ultrastructure analysis, the monolayer culture cells were fixed with 3% glutaraldehyde for 2 hours at 40˚C and post fixed in 1% aqueous osmium tetroxide for 1 hour at 4°C. Fixation was followed by en-block staining in 2% aqueous uranyl acetate. The cells were then subsequently dehydrated in increasing grade of alcohol for 10 mins each. The cells were further infiltrated in increasing series of HPMA followed by Epon resin infiltration for 15 mins each and embedded in Epon resin for polymerization at 60°C for 48 hours. The cells from the polymerised Epon sheet were marked and the sheet was cut into small pieces which were re-embedded on the prepolymerized block and allowed to polymerise. The ultrathin sections of 70nm were cut using Leica UC7 ultramicrotome and collected on 150 mesh copper grid. The sections were contrasted with lead citrate and images were acquired using JEM 1400 Plus Transmission Electron Microscope (JEOL, Japan) at 120kV

**2.7.4. Transcriptome Analysis**

Total RNA was isolated from the TBM-02 (p11 and p62) cell line using Quick RNA MiniPrep Plus kit (Zymo Research) as per manufacturer’s protocol. The quality and quantity of the RNA was determined by Nanodrop followed by agilent tape station using high sensitivity RNA screen tape. The RNA -Seq paired end sequencing libraries were prepared using Illumina TruSeq mRNA sample prep kit. The libraries were enriched by limited number of PCR cycles, purified and were checked for quantity and quality. Cluster generation and sequencing was done by loading the PE Illumina libraries onto Novaseq6000 platform. For normal buccal mucosa, the SRA files were downloaded from the SRA database ([SRX19125628](https://www.ncbi.nlm.nih.gov/sra/SRX19125628%5baccn%5d) and SRX19125629) were subsequently converted into raw fastq files using fastq-dump. High quality reads were obtained after removal of adapter sequences, ambiguous reads (reads with unknown nucleotides “N” larger than 5%), and low-quality sequences (reads with more than 10% quality threshold (QV) < 25 phred score) using Trimmomatic (v0.39). The HQ reads were then aligned to the GRCh38 reference genome ([*https://ftp.ensembl.org/pub/release-108/fasta/homo_sapiens/dna/Homo_sapiens.GRCh38.dna.toplevel.fa.gz*](https://ftp.ensembl.org/pub/release-108/fasta/homo_sapiens/dna/Homo_sapiens.GRCh38.dna.toplevel.fa.gz)), using STAR (v 2.7.10a) at default parameters. featureCounts (v 2.0.3) [3] was employed to obtain the Mapped read counts for individual genes. Differentially expressed genes (DEG) between Cancer cell line (TBM-02 p11 and TBM-02 p62) and the normal buccal mucosa were identified using DESeq2 R package (v 1.40.2). The read counts were normalized for all the samples and differentially expressed Ensembl gene IDs were identified by using the DESeq2 package. Log2 Fold Change (log2FC) values greater than zero were considered up-regulated whereas less than zero were down-regulated along with P-value threshold of 0.05 for statistically significant results. Heatmap plot was constructed using pHeatmap R package considering the log-transformed and normalized value of genes based on Pearson uncentered distance and average linkage method. ClusterProfiler (version 4.2.2) was employed for functional enrichment analysis of KEGG pathways and GO domains using enrichKEGG and enrichGo functions respectively. Pathways and GO terms with p value less than 0.05 were considered significant.

- - 1. **Evaluation of tumorigenicity potential**
       1. **Xenograft Induction and characterization**

NOD-SCID mice (Nonobese diabetic/ Severe combined immunodeficient mice), maintained in Laboratory animal facility (ACTREC) were used for *in vivo* tumorigenesis studies. All procedures involving mice were performed according to protocols approved by the Institutional Animal Ethics Committee, ACTREC, Tata Memorial Centre, Navi Mumbai (Proposal # 16/2021 and 23/2024) and were adhered to CPCSEA guidelines (Registration Number: 65/GO/ReBiBt/S/99/CPCSEA). Animals were randomized before grouping. Animals received humane care and all efforts were undertaken to minimize animal suffering before and during the experiments. 6-8 weeks old male mice weighing 18-22 grams were used for all the experiments. Mice were monitored every three days for body weight, tumor volume, and mortality. Tumor volume was calculated using the formula for the volume. TBM-02 cell suspension was injected subcutaneously into the flank of the mice (n=2) using 1 ml syringes with 25G needles. Mice were observed for tumor for 40 days. The tumor size was assessed by measuring the tumor dimension using a vernier calliper as soon as they reached a measurable size. The tumor volume was calculated using the formula w1*w1*w2*π/6 (w1- smallest width, w2- largest width)

**2.9. Development of *In vitro* gene silencing model:**

**2.9.1. Generation of lenti-viral particles:**

The production of lentiviral particles expressing pLKO.1 constructs adhering to stringent safety protocols approved by the institutional biosafety committee of ACTREC-TMC. All operations were conducted within a certified biosafety level 2 laminar flow cabinet (Esco Technologies, Hatboro, PA, USA). Following procedures, infected cell cultures, contaminated plasticware, and fluids underwent thorough decontamination using a 10% sodium hypochlorite solution before being autoclaved in biohazard bags for disposal. The HEK293FT cell line served as the packaging host for lentiviral particle generation, utilizing second-generation packaging plasmids psPAX2 (coat) and pMD2.G (envelope), both sourced from Addgene (psPAX2, plasmid #12260; pMD2.G, plasmid #12259, generously provided by Dr. Didier Trono). Additionally, the pAdvantage vector (Promega, Madison, WI, USA) was employed to bolster the transient protein expression efficiency within host cells.

- - 1. **Transduction with lentiviral particles for stable expression:**

HEK293FT cells were seeded at a density of 6-7 x 10^5^ cells in 60 mm culture dishes the day before transfection with the aim of reaching 50-60% confluency at the time of transfection. Four hours prior to transfection, the spent medium was replaced with fresh complete medium. For transfection, a total of 12 µg of plasmid mixture containing transfer, coat, and envelope plasmids in a 4:3:1 ratio (6 µg of Transfer plasmid pLKO_005 shRNA NLRP3/ pLKO.1 Scrambled, 4.5 µg coat plasmid psPAX2, and 1.5 µg envelope plasmid pMD2.G) along with 3 µg pAdvantage was prepared in a sterile 1.5 ml centrifuge tube and diluted to 100 µl with sterile ultrapure water. Subsequently, 100 µl of 0.5 M CaCl_2_ solution was added dropwise to the plasmid mixture, followed by the addition of 200 µl of 2X BES Buffered Saline (2X BBS) solution to form a total of 400 µl of the transfection mixture. After gentle mixing, the mixture was incubated at room temperature for 20 min. The DNA-calcium phosphate complexes were then added dropwise to the HEK293FT cells, and the cells were incubated at 37°C in a CO_2_ incubator. Fresh complete medium replacement was done 16-18 h post-transfection. Lentivirus-containing cell culture supernatant was collected at 48 h and 72 h post-transfection, centrifuged, and filtered. The filtered supernatant was either directly used for transduction or concentrated by ultracentrifugation. Transduced cells were selected using puromycin for 8 days. Thereafter it was further expanded using IMDM containing 10% FBS supplementing with 1% Penicillin and Streptomycin.
