# Supplementary material for: Generation and characterization of early stage oral cancer cell line of buccal mucosa of Indian origin
Source: Hum Cell. 2025 Dec 18;39(1):21. doi: 10.1007/s13577-025-01332-6 (PMC12714774; doi:10.1007/s13577-025-01332-6)
Supplement: Supplementary file 2 — (DOCX 701 KB) [file 13577_2025_1332_MOESM2_ESM.docx]

**Generation and characterization of Early-stage Oral Cancer cell line of Buccal mucosa of Indian Origin**

Akhila George^1,2^, Sudhir Nair ^2,3,4^, Kumar Prabhash^2,4,5,6^, Sayujata Thakur^1^, Poonam Gera^7^, Arjun Singh^2,3,4^, Pankaj Chaturvedi^2,3,4^, Swapnil Rane^2,4,8^, Trupti Pradhan^1^, Subrata Sen^9^, Madan Barkume^9^, Dhanlaxmi Shetty^2^,^10^, Kruti Chaubal^10^, Arpita Ghosh^11^, Sanjeev Kamte^11^, Jyoti Kode^1,2,9*^

Supplementary figures:


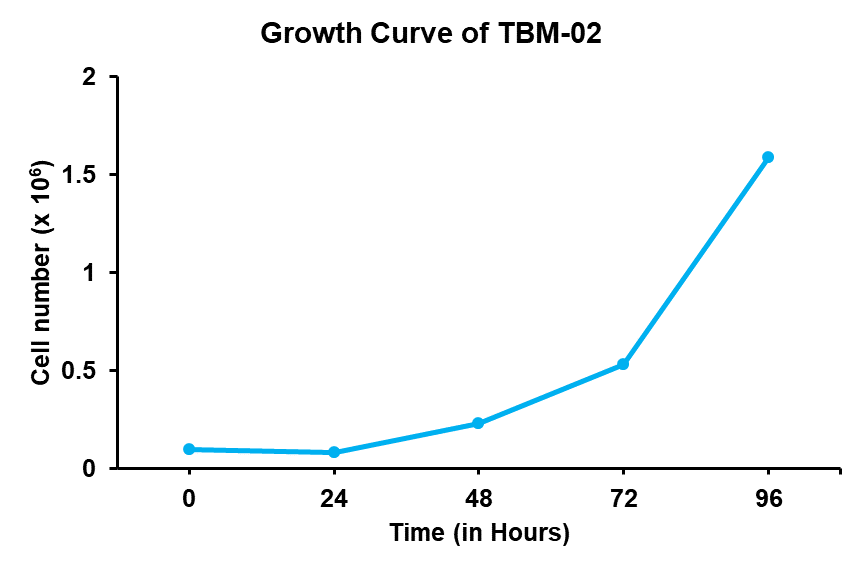


Figure S1: Growth curve of Cell line TBM-02; Doubling time of the cell line was found to be 26.39 hours.


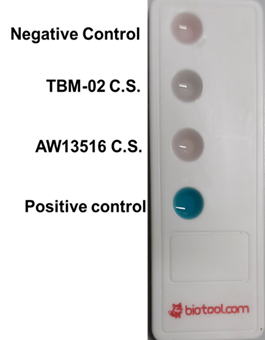


Figure S2: Mycoplasma detection in cell culture supernatant; the cell culture supernatant of TBM-02 shows absence of any mycoplasma contamination

Figure S3: STR profile of TBM-02 at passage 50.


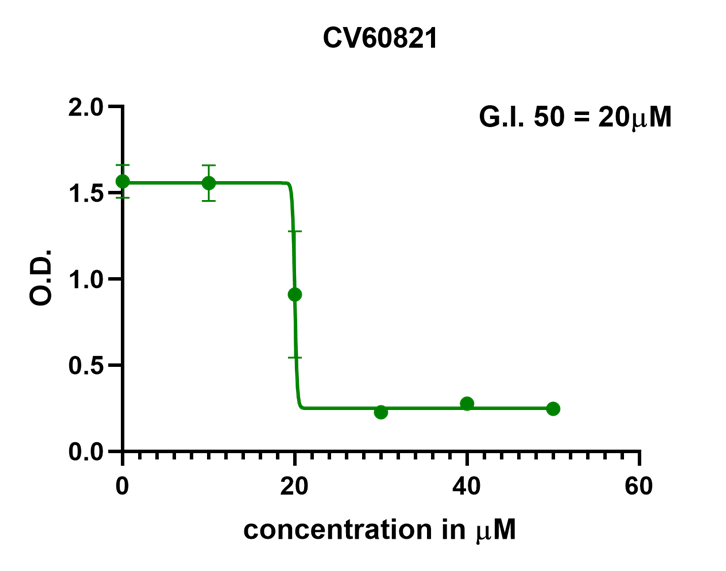


Figure S4: Inhibitory concentration (IC_50_) of phytochemical 6-Shogaol was found to be 20 μM


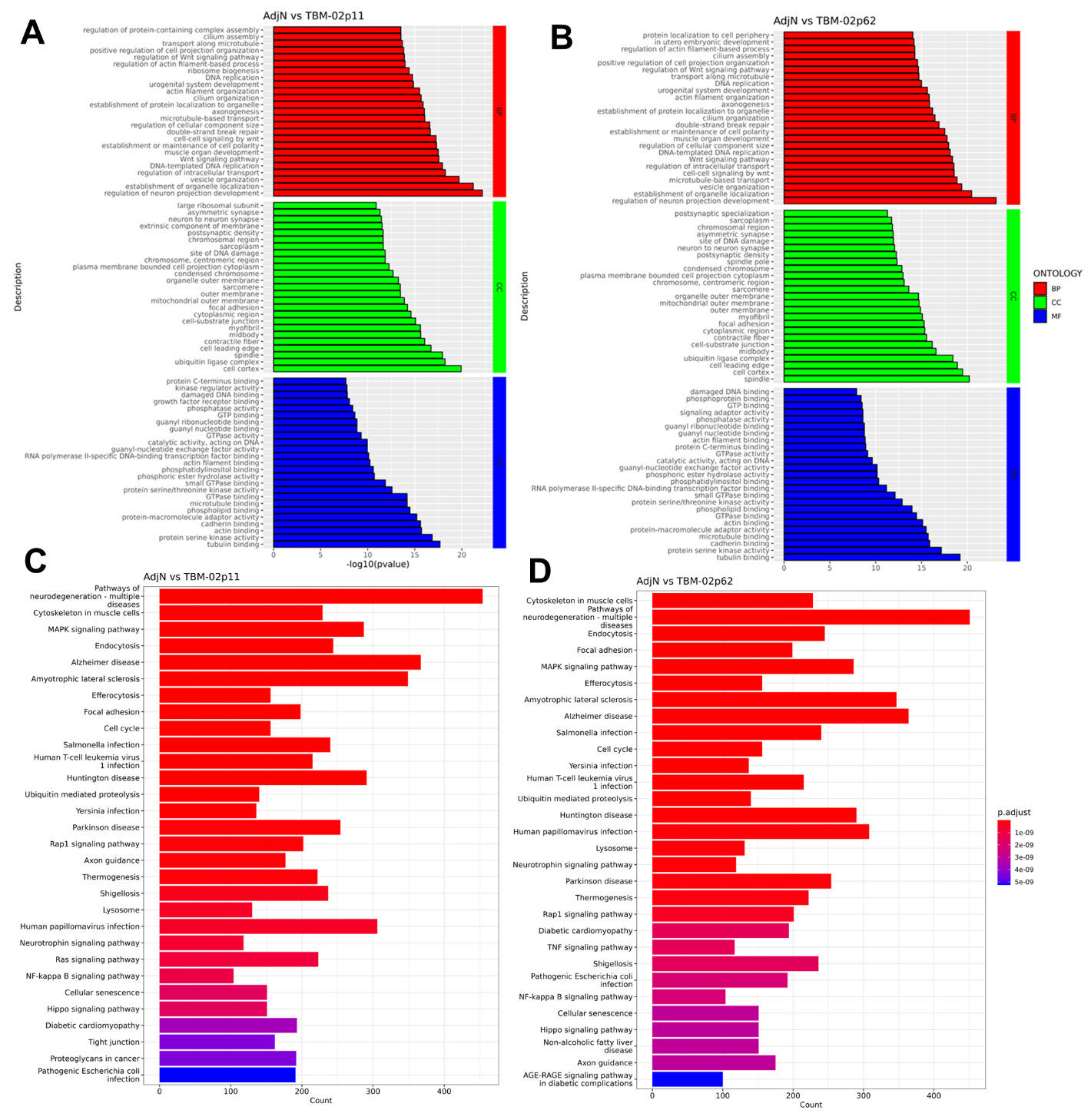


**Figure S4: Transcriptome analysis of TBM-02 cell line**. The graphs show gene ontology to decipher the genes associated with the dysregulated pathways of TBM-02 at early passage p11(A) and late passage p62 (B). KEGG pathways analysis in TBM-02 p11 (C) and p62 (D) in comparison to normal buccal mucosa (SRA database [SRX19125628](https://www.ncbi.nlm.nih.gov/sra/SRX19125628%5baccn%5d) and SRX19125629).
