# Supplementary material for: Generation and characterization of early stage oral cancer cell line of buccal mucosa of Indian origin
Source: Hum Cell. 2025 Dec 18;39(1):21. doi: 10.1007/s13577-025-01332-6 (PMC12714774; doi:10.1007/s13577-025-01332-6)
Supplement: Supplementary file 3 — (DOCX 23 KB) [file 13577_2025_1332_MOESM3_ESM.docx]

**Generation and characterization of Early-stage Oral Cancer cell line of Buccal mucosa of Indian Origin**

Akhila George^1,2^, Sudhir Nair ^2,3,4^, Kumar Prabhash^2,4,5,6^, Sayujata Thakur^1^, Poonam Gera^7^, Arjun Singh^2,3,4^, Pankaj Chaturvedi^2,3,4^, Swapnil Rane^2,4,8^, Trupti Pradhan^1^, Subrata Sen^9^, Madan Barkume^9^, Dhanlaxmi Shetty^2^,^10^, Kruti Chaubal^10^, Arpita Ghosh^11^, Sanjeev Kamte^11^, Jyoti Kode^1,2,9*^

**Supplementary Table S1: Details of reagents, cell lines and animals used in the study**

| **Sr No.** | **Reagent name** | **Company** | **Catalogue no.** | **Conjugate** |
| --- | --- | --- | --- | --- |
| **1.** | **Reagents** |  |  |  |
| a | IMDM | Gibco, India | 12200-036 |  |
| b | FBS | Gibco | F2442 |  |
| c | DMSO | M.P. Biochemicals | 0219605590 |  |
| d | Antibiotic Antimycotic solution | Himedia | A002A |  |
| e | Enzyme cocktail |  |  |  |
|  | DNAse | Sigma | D5025 |  |
|  | Collagenase | Sigma | C5138 |  |
|  | Hyaluronidase | Sigma | H3506 |  |
| f | Wokadine | Wockhart limited |  |  |
| g | Trypan blue | Sigma | T6146 |  |
| h | Colchicine | Sigma-Aldrich | C9754 |  |
| i | Trypsin | Sigma-Aldrich | T4049 |  |
| j | Paraformaldehyde | Sigma | P6148 |  |
| k | Sodium azide | Sigma | 26628-22-8 |  |
| l | Lipopolysaccharide | Sigma | L-2880 |  |
| m | Nigericin | Sigma | N-7143 |  |
| n | Crystal violet | Sigma-Aldrich | C3886 |  |
| o | MCC 950 | Sigma-Aldrich | 5281200001 |  |
|  |  |  |  |  |
| **2.** | **Apparatus** |  |  |  |
| a | Boyden chamber | Himedia | TCP257-4X12NO |  |
|  |  |  |  |  |
| **3.** | **Mycoplasma detection kit** | **Biotool.com** | **B39038** |  |
|  |  |  |  |  |
| **4.** | **Cell growth estimation** |  |  |  |
| a | SRB | Sigma | S9012 |  |
|  |  |  |  |  |
| **5.** | **Immunohistochemistry** |  |  |  |
| a | Envision FLEX Mini Kit | Dako | K-8023 |  |
| b | IL-18 | Abcam | Ab243091 |  |
| c | NLRP3 | Invitrogen | MA5-32255 |  |
| d | Caspase-1 | Invitrogen | MA5- 16215 |  |
| e | GSDMD | Sigma-Aldrich | SAB4200798 |  |
|  |  |  |  |  |
| **6.** | **Immunoflouoresence** |  |  |  |
| a | NLRP3 | Invitrogen | MA5-32255 |  |
| b | α -Tubulin | Abcam | Ab7291 |  |
| c | Keratin 8 | Novus | Nbp216094 |  |
| d | Keratin 14 | Abcam | Ab7800 |  |
| e | Goat anti-Mouse AF-488 | Invitrogen | A-11001 |  |
| f | Goat anti- rabbit AF-568 | Invitrogen | A-11011 |  |
|  |  |  |  |  |
| **7**. | **Immunophenotyping** |  |  |  |
| a | EpCAM | B.D. Biosciences | 563180 | BV 421 |
| b | α – SMA (D4K9N) | Cell Signalling Technologies | #19245 |  |
|  |  |  |  |  |
| **8.** | **Drugs** |  |  |  |
| a | Gemcitabine | Bruck-Gemtrox |  |  |
| b | Cisplatin | Bruck-Platicys |  |  |
| c | Carboplatin | Bruck-Carbonco |  |  |
| d | Mitoxantrone | Neon-Nitrol |  |  |
| e | Etoposide | Oncocare-Posid |  |  |
| f | Oxaliplatin | Bruck-Oxatoxin |  |  |
| g | Vinblastine | Zuvius-Z-Blastin |  |  |
| h | Paclitaxel | Emyrus-Emytaxel |  |  |
| i | Doxorubicin | Bruck-Adridox |  |  |
| j | 6-Shogaol | Sigma | 39303 |  |
|  |  |  |  |  |
| **9** | **Cell cycle** |  |  |  |
| a | RNase A | Sigma | R6513 |  |
| b | Propidium Iodide | Sigma | 4170 |  |
|  |  |  |  |  |
| **10** | **STR PowerPlex kit** | Promega |  |  |
|  |  |  |  |  |
| **11** | **Real-Time PCR** |  |  |  |
|  | DNAse kit | Thermoscientific | EN0525 |  |
|  | RevertAid H minusfirst strand cDNA synthesis kit | Thermoscientific | K1632 |  |
|  | KAPA SYBR Green | KAPA Biosystems | KK4602 |  |
|  | TRIzol | Invitrogen | 15596026 |  |
|  |  |  |  |  |
| **12** | **Plasmids** |  |  |  |
| a | pLKO-005 | Merck (sigma Aldrich) | TRCN0000419896 |  |
| b | psPAX2 | Addgene | 12260 |  |
| c | pMD2.G | Addgene | 12259 |  |
| d | pAdvantage | Promega |  |  |
|  |  |  |  |  |

**Supplementary Table S2: Primer Sequences**

| 18s Forward | 5’ GAT GGT AGT CGC CGT GCC 3’ |
| --- | --- |
| 18s Reverse | 5’ GCC TGC TGC CTT CCT TGG 3’ |
| NLRP3 Forward | 5’- ATG AGC CGA AGT GGG GTT -3’ |
| NLRP3 Reverse | 5’- GTG TGT AGC GTT TGT TGA GG -3’ |

**Supplementary Table S3: Clinicopathological features of the patient**

| Patient Code | P2 |
| --- | --- |
| Age | 34 |
| Sex | Male |
| Habits | Tobacco chewer |
| Site | Right Buccal Mucosa |
| TNM Staging | pT2 pN0 |
| Perineural Invasion | Absent |
| Depth of invasion | 0.9 cm |
| Predominant pattern of Invasion | Type 3 |
| Worst pattern of Invasion | Type 4 |
| Patient status | Alive |

**Supplementary Table S4:** **Early-stage tumor associated genes expressed differentially in TBM-02**

| **Sr.No.** | **Gene** | **Regulation** | **Log2fold change p11** | **Log2fold change p62** |
| --- | --- | --- | --- | --- |
| 1* | FAM3B | Downregulated | -5.36 | -4.65 |
| 2 | ANGPTL5 | Downregulated | -7.66 | -4.93 |
| 3 | GREM2 | Downregulated | -7.61 | -10.76 |
| 4 | ESRRG | Downregulated | -10.63 | -3.56 |
| 5 | INHBA | Upregulated | 1.56 | 0.66 |
| 6 | TP53 | Upregulated | 2.76 | 1.86 |
| 7 | BRD4 | Downregulated | -0.05 | -0.23 |
| 8 | VEGFA | Upregulated | 0.83 | 0.26 |
| 9 | MKI67 | Upregulated | 1.72 | 3.68 |
| 10 | MYC | Upregulated | 1.49 | 0.74 |
| 11** | ACP5 | Downregulated | -6.66 | -6.82 |
| 12 | SPP1 | Downregulated | -6.92 | -7.39 |
| 13 | MMP12 | Downregulated | -14.20 | -11.51 |

*Genes Sr.No. 1-10 were selected from our own patient data of genes expressed differentially in early-stage tumor versus corresponding adjacent normal. The data is provided from differential comparison of normal buccal mucosa (SRA database [SRX19125628](https://www.ncbi.nlm.nih.gov/sra/SRX19125628%5baccn%5d) and SRX19125629) versus TBM-02 cell line at early passage 11 (p11) and late passage 62 (p62).

** Genes Sr.No. 11-13 were selected from our own patient data of genes expressed differentially in late-stage tumor versus corresponding adjacent normal. The data is provided from differential comparison of normal buccal mucosa (SRA database [SRX19125628](https://www.ncbi.nlm.nih.gov/sra/SRX19125628%5baccn%5d) and SRX19125629) versus TBM-02 cell line at early passage 11 (p11) and late passage 62 (p62).
